# Comprehensive Summary of Safety Data on Nirsevimab in Infants and Children from All Pivotal Randomized Clinical Trials

**DOI:** 10.3390/pathogens13060503

**Published:** 2024-06-13

**Authors:** Vaishali S. Mankad, Amanda Leach, Yue Chang, Ulrika Wählby Hamrén, Alexandre Kiazand, Robert J. Kubiak, Therese Takas, Tonya Villafana, Manish Shroff

**Affiliations:** 1 Vaccines & Immune Therapies, BioPharmaceuticals R&D, AstraZeneca, Durham, NC 27703, USA; vaishali.mankad@astrazeneca.com; 2 Vaccines & Immune Therapies, BioPharmaceuticals R&D, AstraZeneca, Gaithersburg, MD 20878, USA; amandaleach667@gmail.com (A.L.); yue.chang1@astrazeneca.com (Y.C.); therese.takas@astrazeneca.com (T.T.); tonya.villafana@astrazeneca.com (T.V.); 3 Clinical Pharmacology and Quantitative Pharmacology, R&D, AstraZeneca, SE-43183 Gothenburg, Sweden; ulrika.wahlby-hamren@astrazeneca.com; 4 Patient Safety, Chief Medical Office, Oncology R&D, AstraZeneca, Gaithersburg, MD 20878, USA; alexandre.kiazand@astrazeneca.com; 5 Clinical Pharmacology and Quantitative Pharmacology, BioPharmaceuticals R&D, AstraZeneca, Gaithersburg, MD 20878, USA; robert.kubiak@astrazeneca.com; 6 Patient Safety, Chief Medical Office, Oncology R&D, AstraZeneca, Waltham, MA 02451, USA

**Keywords:** infants, monoclonal antibody, nirsevimab, respiratory syncytial virus, safety

## Abstract

Background: Nirsevimab is approved in the US for the prevention of respiratory syncytial virus (RSV) lower respiratory tract disease in neonates and infants during their first RSV season and in children aged ≤24 months who remain vulnerable to severe RSV disease through their second RSV season. We summarize a pre-specified analysis of nirsevimab safety data from three randomized controlled trials: Phase 2b (NCT02878330; healthy infants born ≥29 to <35 weeks’ gestational age [wGA]); Phase 3 MELODY (NCT03979313; healthy infants born ≥35 wGA); and Phase 2/3 MEDLEY (NCT03959488; infants with congenital heart disease [CHD] and/or chronic lung disease of prematurity [CLD] or born ≤35 wGA). Methods: Participants (randomized 2:1) received a single intramuscular dose of nirsevimab or comparator (placebo, Phase 2b/MELODY; 5× once-monthly palivizumab, MEDLEY) before their first RSV season (recipients < 5 kg, nirsevimab 50 mg; ≥5 kg, nirsevimab 100 mg). In MEDLEY, children with CHD/CLD continued to a second RSV season: first-season nirsevimab recipients received nirsevimab 200 mg; first-season palivizumab recipients were re-randomized 1:1 to receive nirsevimab 200 mg or 5× once-monthly palivizumab. Results: The incidence, severity, and nature of AEs were similar across treatments (nirsevimab, *n* = 3184; placebo, *n* = 1284; palivizumab, *n* = 304). Most AEs were mild to moderate in severity, with ≥98% unrelated to treatment. AEs of special interest occurred infrequently (<1%): no anaphylaxis or thrombocytopenia were treatment-related, and no immune complex disease was reported. Deaths (incidence < 1.0%) were all unrelated to treatment. Conclusions: A single dose per season of nirsevimab for the prevention of RSV disease had a favorable safety profile, irrespective of wGA or comorbidities.

## 1. Introduction

Respiratory syncytial virus (RSV) is the most common cause of lower respiratory tract infection (LRTI) in infants globally and the most frequent cause of hospitalization in the first year of life [1,2]. Healthy term infants account for most of the disease burden and hospitalizations due to RSV LRTI, yet historically, they have been ineligible to receive preventative measures against RSV disease [3].

In early trials of active vaccination in the 1960s, infants received a formalin-inactivated whole RSV vaccine (FI-RSV) [4,5]. Although an active immune response was observed, following subsequent natural exposure to RSV, children who were previously seronegative to RSV developed enhanced respiratory disease (ERD), leading to hospitalization rates of 80% and two deaths among FI-RSV recipients, compared with hospitalization rates of 15% and no deaths among placebo recipients [5]. The failure of this experimental vaccine led to the development of a passive immunization approach with palivizumab, a humanized monoclonal antibody (mAb) comprising 95% human and 5% murine sequences [6]. Palivizumab improved upon the polyclonal RSV hyperimmune globulin derived from pooled plasma donors and enriched for RSV-neutralizing antibodies [7,8] and is indicated for infants at higher risk of serious RSV lower respiratory tract disease [6], including those with congenital heart disease (CHD), those with chronic lung disease of prematurity (CLD), or those born prematurely [1,9,10]; it is administered via a monthly intramuscular (IM) injection throughout the RSV season.

Nirsevimab is a human IgG1 kappa neutralizing mAb against an epitope unique to the prefusion conformation of the RSV fusion (F) protein [11]. Following natural RSV infection, most neutralizing activity is directed at the prefusion form of the F protein [12], thereby making it a better target for mAb development than the postfusion form. Nirsevimab works by binding and locking the RSV F protein in the prefusion conformation, thereby preventing the refolding necessary for the essential membrane fusion step required to initiate virus entry and, therefore, cell infection [11,13]. Moreover, nirsevimab has an engineered triple amino acid substitution, M252Y/S254T/T256E, in the Fragment crystallizable (Fc) region that enhances the binding of the Fc region to the neonatal Fc receptor (FcRn), thereby increasing FcRn recycling and protecting nirsevimab from proteolytic degradation [14]. This modification leads to an approximately 3-fold increase in nirsevimab serum antibody half-life, from 21–28 days [15] to approximately 70 days, in healthy late-preterm and term infants [11,16,17].

The safety and efficacy of a single dose of nirsevimab administered before an RSV season was assessed in three global, randomized, double-blind clinical trials. The Phase 2b (NCT02878330) [18] and Phase 3 MELODY trials (NCT03979313) [16,19] evaluated the safety and efficacy of a single dose of nirsevimab in otherwise healthy preterm and term infants, while the Phase 2/3 MEDLEY trial (NCT03959488) evaluated the safety and pharmacokinetics of nirsevimab in infants eligible to receive palivizumab before their first RSV season (preterm infants) or before each of two consecutive RSV seasons (children with CHD/CLD) [20,21]. Nirsevimab was found to have a favorable safety profile, and efficacy was demonstrated to be 70.1–79.5% [16,18,19,22]. Nirsevimab was subsequently approved in the US for the prevention of RSV lower respiratory tract disease in all neonates and infants during their first RSV season and in children up to 24 months of age who remain vulnerable to severe RSV disease through their second RSV season (i.e., those with CHD/CLD) [23].

This report aims to comprehensively summarize the safety of nirsevimab for the prevention of RSV lower respiratory tract disease in infants and children < 24 months of age from pivotal trials.

## 2. Methods

Designs for Phase 2b, MELODY, and MEDLEY have been detailed previously [16,18,20]. Healthy term and preterm infants in Phase 2b (born ≥29 to <35 weeks’ gestational age [wGA]) and MELODY (born ≥35 wGA) were randomized 2:1 to receive a single IM dose of nirsevimab or placebo before their first RSV season [16,18]. A weight-banded regimen was adopted in MELODY and MEDLEY (participants < 5 kg: 50 mg; ≥5 kg: 100 mg nirsevimab) following pharmacokinetic and drug exposure–response analyses from Phase 2b [16,18,20,22].

In MEDLEY, infants at higher risk of severe RSV disease entering their first RSV season and eligible to receive palivizumab who were either born ≤35 wGA without CHD or CLD or who had uncorrected, partially corrected, or medically treated CHD and/or CLD warranting therapeutic intervention within 6 months were randomized 2:1 to receive a single IM weight-banded nirsevimab dose followed by 4× once-monthly IM placebo doses or, alternatively, 5× once-monthly IM palivizumab doses (15 mg/kg of body weight per dose) [20]. In the second season, participants with CHD/CLD randomized to nirsevimab in the first season received a single IM dose of nirsevimab 200 mg, followed by 4× once-monthly IM placebo doses (nirsevimab/nirsevimab), while participants randomized to palivizumab were re-randomized to receive either a single IM dose of nirsevimab 200 mg followed by 4× once-monthly IM placebo doses (palivizumab/nirsevimab) or 5× once-monthly IM palivizumab doses (palivizumab/palivizumab) [21]. Participants who underwent cardiac surgery with cardiopulmonary bypass after receiving their first treatment dose, but prior to their fifth dose in either season, received a replacement injection immediately following surgery.

In this pre-specified analysis, safety findings are presented using pooled data from participants in the Phase 2b study [18] (conducted 2016–2018) and the full MELODY enrollment cohort [19] through the final safety follow-up (conducted 2019–2023) who received the approved, weight-banded dose of nirsevimab (healthy term and preterm infants ≥ 29 wGA) as well as data from both seasons of MEDLEY through the final database lock on 22 February 2023 (conducted 2019–2023) [20,21]. Across all studies and seasons, unsolicited treatment-emergent adverse events (AEs) were captured through 360 days post-dose. Additional details on AE assessments in MEDLEY are described in Supplementary Section S1.

AEs were graded by severity according to the National Cancer Institute Common Terminology Criteria for Adverse Events, version 5.0, and coded according to the Medical Dictionary for Regulatory Activities, version 25.1. AEs of special interest (AESIs) comprised type I hypersensitivity (including anaphylaxis), immune complex disease, and thrombocytopenia. Results are reported for the as-treated populations and summarized with descriptive statistics; subgroups of interest are described in Supplementary Section S2.

## 3. Results

### 3.1. Participants and Treatment Exposure

Overall, 3184 infants received the approved nirsevimab dose, 1284 received placebo, and 304 received palivizumab before or during their first RSV season (Table 1; Supplementary Figures S1 and S2). Baseline demographics and clinical characteristics were balanced between treatment groups (Table 1). Primary cardiac lesions for participants with CHD were reported previously [20,21].

In Phase 2b and MELODY, 2570 healthy term and preterm infants received the approved nirsevimab dose, while 1284 received placebo. In MEDLEY, 612 preterm infants received nirsevimab (*n* = 406) or palivizumab (*n* = 206), and 306 infants with CHD/CLD received nirsevimab (*n* = 208) or palivizumab (*n* = 98). Before their second RSV season, 180 children with CHD/CLD received a second nirsevimab dose. Of those who received palivizumab in the first season, 42 received palivizumab and 40 received a first dose of nirsevimab in the second season. All nirsevimab recipients received ≥1 active dose, and >90% of palivizumab recipients received ≥5 active doses; >85% of participants in each study and season completed 360 days of safety follow-up (Supplementary Table S1).

### 3.2. Overall Summary of AEs

**Figure 1:**
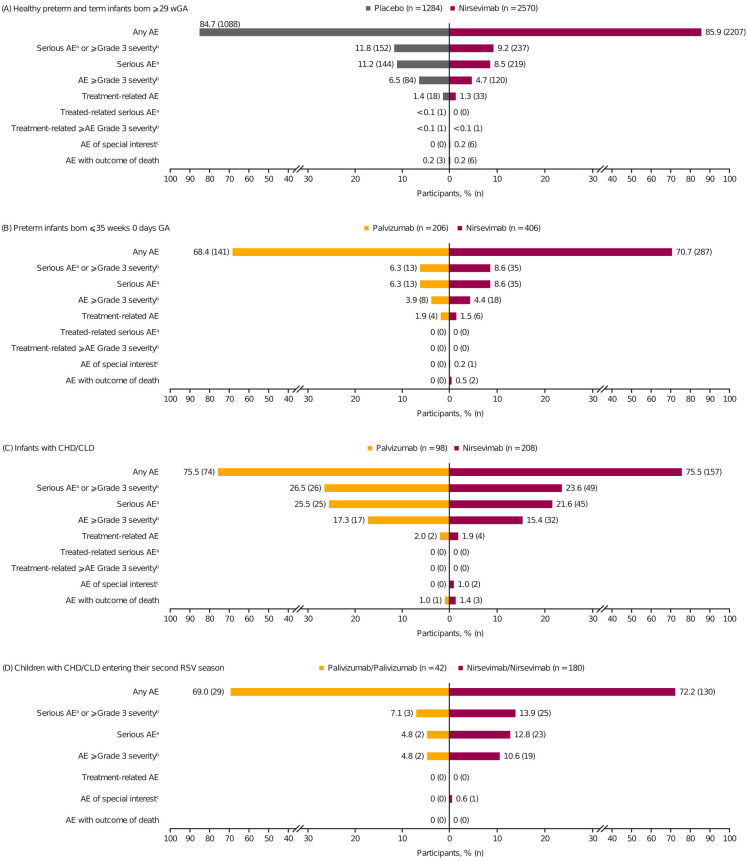
Overall summary of AEs through 360 days post-dose in (**A**) healthy term and preterm infants ≥ 29 wGA (includes infants from Phase 2b weighing < 5 kg and the full MELODY enrollment cohort) and palivizumab-eligible infants at higher risk of severe RSV disease from the MEDLEY trial, including (**B**) preterm infants ≤ 35 weeks 0 days GA without CLD or CHD, (**C**) infants with CHD/CLD (all entering their first RSV season), and (**D**) children with CHD/CLD entering their second RSV season (before the second season, children with CHD/CLD randomized to nirsevimab in the first season received a single IM dose of 200 mg nirsevimab followed by 4× once-monthly IM doses of placebo [nirsevimab/nirsevimab], and those randomized to palivizumab in the first season were re-randomized 1:1 to either a single IM dose of 200 mg nirsevimab followed by 4× once-monthly IM doses of placebo [palivizumab/nirsevimab] or 5× once-monthly IM doses of palivizumab [15 mg/kg per dose; palivizumab/palivizumab]). Participants with multiple events in the same category were counted once in that category; participants with events in >1 category were counted once in each category. (a) Defined as death, life-threatening, requiring inpatient hospitalization, prolongation of existing hospitalization, persistent or significant disability/incapacity, important medical event, or congenital anomaly/birth defect. (b) Grade 1: mild; Grade 2: moderate; Grade 3: severe; Grade 4: life-threatening; Grade 5: fatal. (c) Included immediate type I hypersensitivity reactions (including anaphylaxis), immune complex disease, and thrombocytopenia. Abbreviations: AE, adverse event; CHD, congenital heart disease; CLD, chronic lung disease of prematurity; GA, gestational age; IM, intramuscular; RSV, respiratory syncytial virus; wGA, weeks’ gestational age.

Through 360 days post-dose, the overall AE, serious AE, and treatment-related AE incidences were generally balanced among infants who received any treatment before their first RSV season (Figure 1a–c), including among infant subgroups of interest (Supplementary Figure S3a–c). The AE, serious AE, and treatment-related AE incidences within 30 days of the first dose were similar between nirsevimab and palivizumab among preterm infants and infants with CHD/CLD (Supplementary Figure S4a,b) and among children with CHD/CLD who received nirsevimab or palivizumab in consecutive RSV seasons (Supplementary Figure S3d,e), with a similar AE profile observed between treatment groups within 30 days of first dosing in the second season (Supplementary Figure S4c). Palivizumab recipients in the first season and nirsevimab recipients in the second season had similar AE incidences versus those who received palivizumab in consecutive RSV seasons (Supplementary Figure S5). Across studies, most AEs were mild or moderate in severity and unrelated to treatment, with no relationship between nirsevimab serum exposure (assessed by area under the time–concentration curve from Day 0 to 365 or maximum serum concentration) and the occurrence of serious or ≥Grade 3 AEs or AESIs (Supplementary Figure S6).

A single preterm nirsevimab recipient was discontinued from treatment due to a related hypersensitivity AESI temporally associated with receiving placebo (3 months after the active nirsevimab dose; Supplementary Table S2) but remained in the study. There were no study discontinuations due to an AE among dosed participants across trials.

### 3.3. Most Common AEs

The most commonly observed Aes were similar between nirsevimab and comparator recipients (Table 2) and consistent with expectations for the respective study populations. The most common Aes within 14 days post-dosing were consistent between nirsevimab and placebo recipients in healthy preterm and term infants, irrespective of co-administered routine childhood vaccinations (Supplementary Table S3).

### 3.4. Treatment-Related AEs

The incidence of treatment-related AEs was ≤2.0% across all trials (Figure 1); those occurring at a higher frequency among nirsevimab versus comparator recipients included injection site reactions and rashes (Supplementary Table S4). There were no treatment-related AEs reported in the second season following nirsevimab or palivizumab treatment, regardless of the treatment received in the first season. The percentage of participants with post-baseline antidrug antibodies (ADAs) anytime through Day 361 (Supplementary Table S5) was low. Nirsevimab recipients who were positive for ADAs post-baseline had a similar safety profile compared with those who were negative for ADAs post-baseline and/or comparator groups (Supplementary Tables S6 and S7).

### 3.5. Measures of Reactogenicity

Systemic and local reactogenicity measures, typically solicited within 7 days post-dose in trials of vaccines in infants and children, occurred in <1% of healthy preterm and term infants (pyrexia: nirsevimab, 13 [0.5%], placebo, 8 [0.6%]; injection site reactions: nirsevimab, 7 [0.3%], placebo, 0; Supplementary Table S8).

### 3.6. AEs of Special Interest

Across all studies and seasons, the incidence of AESIs was low (<1.0%; Figure 1, Supplementary Figure S5a). The case details of AESIs of hypersensitivity were generally inconsistent with immediate hypersensitivity, and no anaphylaxis was attributed to nirsevimab or its comparators (Supplementary Table S2). No ADAs were detected in participants with hypersensitivity, apart from a single participant with a maculopapular rash on Day 1 for whom ADAs were first detected on Day 361 (Supplementary Table S2). No participants in MEDLEY who received a replacement dose of nirsevimab following cardiopulmonary bypass (eight participants in Season 1; two participants in Season 2) had post-baseline ADAs or AESIs of hypersensitivity through Day 361. An AESI of thrombocytopenia was observed in participants with CHD and other significant underlying comorbidities following heparin administration (one participant) or infections (two participants) and was not considered related to nirsevimab (Supplementary Table S2). No immune complex disease was reported in any trial.

### 3.7. Serious AEs and AEs with Outcome of Death

The most commonly observed serious AEs were consistent across treatment groups in the first RSV season, with the vast majority resulting from infections (Table 3). Following second RSV season dosing, the incidence of serious AEs and AEs ≥ Grade 3 severity was numerically higher among nirsevimab recipients (nirsevimab/nirsevimab and palivizumab/nirsevimab groups) than palivizumab/palivizumab recipients (Figure 1D, Supplementary Figure S5a); these events were primarily due to infections or related to underlying comorbid conditions (Supplementary Tables S9 and S10), and most occurred >30 days after dosing (Supplementary Figure S5a,b); no trends or safety concerns were identified.

The incidence of AEs leading to death was low across studies (<1.0%), and none were considered treatment-related (Figure 1). Causes of death were attributed to underlying medical conditions or causes of infant mortality common to the region where the participants were enrolled (Supplementary Tables S11 and S12). Among healthy preterm and term infants, the incidence of death was low and balanced between nirsevimab (0.2%) and placebo recipients (0.2%). Among preterm infants, the incidence of death was <1% in both nirsevimab and palivizumab recipients and 1.4% and 1.0%, respectively, among infants with CHD/CLD. These deaths occurred in infants with serious underlying medical conditions at baseline; none were considered related to nirsevimab. No deaths were reported among children dosed in consecutive RSV seasons.

## 4. Discussion

In this comprehensive, pre-specified safety analysis of 3184 infants who received nirsevimab in their first RSV season and 220 children in their second season, nirsevimab showed a favorable safety profile, being well tolerated in healthy term and preterm infants ≥ 29 wGA and palivizumab-eligible children at higher risk of severe RSV disease, including premature infants and those with CHD/CLD. Generally, the incidence, nature, and severity of AEs were similar between treatment groups and consistent with expectations for the respective trial populations. Most AEs were mild or moderate in severity and considered unrelated to nirsevimab. No deaths were considered related to nirsevimab or comparators, and the causes of death varied, with no pattern for the types of fatal AEs.

The safety profile of nirsevimab was generally consistent with that observed in palivizumab trials, where the incidence of treatment-related AEs was similar between palivizumab and placebo recipients, discontinuation due to treatment-related AEs was infrequent (<2%) [24], and no deaths were attributed to palivizumab [25,26].

Since nirsevimab is specific for RSV without any endogenous targets, AESIs comprised risks common to any exogenous immunoglobulin, including hypersensitivity reactions and immune complex disease [27], which could potentially be triggered by ADAs. Consistent with expectations for a fully human mAb, these pre-specified AESIs were uncommon across studies, with no reports of immune complex disease among nirsevimab recipients. Additionally, no anaphylaxis or other serious allergic reactions were attributed to nirsevimab. Rashes reported as events of hypersensitivity by investigators occurred with a low incidence (<1%); none were associated with ADAs, characterized as urticarial, or involved angioedema typical of immediate hypersensitivity. Furthermore, no hypersensitivity was reported following the dosing of nirsevimab in consecutive RSV seasons. This contrasts with motavizumab, an investigational anti-RSV F mAb administered monthly during the RSV season that did not proceed to licensure; here, a threefold increase in reactions suggesting immediate hypersensitivity (e.g., urticaria, edema) was observed versus palivizumab in clinical trials of infants at higher risk for severe RSV disease [28,29]. Anaphylaxis, including fatalities, has been reported following initial or subsequent palivizumab doses, and “anaphylaxis and other acute hypersensitivity reactions” are included among the most serious adverse reactions in the palivizumab prescribing information [6,24].

Post-marketing reports of thrombocytopenia with palivizumab [6,24] prompted the inclusion of thrombocytopenia as an AESI in the nirsevimab clinical development program. Although the mechanism of thrombocytopenia is unclear, and it is challenging to establish causality or estimate the true frequency based on voluntary post-marketing reporting [6,24], in clinical trials of nirsevimab, thrombocytopenia events were nonserious and considered unrelated to treatment. Ultimately, the events were attributed to established, alternate causes, including heparin [30], sepsis [31], and roseola (exanthem subitem) with aseptic meningitis due to confirmed Human Herpes Virus 6 infection [32].

Reactogenicity, a term typically used in the context of vaccination, is defined as the inflammatory response mediated by the innate immune system recognizing foreign antigens soon after vaccination, resulting in local (e.g., injection site reactions) and systemic (e.g., fever) clinical manifestations [33]. As nirsevimab is a fully human mAb for passive immunization specifically against RSV, making it less likely to trigger an innate immune response, the incidence of AEs indicative of reactogenicity measures typically solicited in pediatric vaccine trials was low and consistent with the expected potential for reactogenicity. This contrasts with higher reactogenicity rates observed following routine childhood vaccines, particularly for fever [34,35], and would suggest that the co-administration of nirsevimab with routine childhood vaccines would not result in an additive adverse reaction or interfere with the immune response to co-administered vaccines [36]. Indeed, in healthy term and preterm infants, we saw no effect of co-administration of routine childhood vaccines on the safety profile of nirsevimab. As reactogenicity assessments in this analysis were based on reported AEs and were not actively solicited, this could lead to an underestimation of rates; however, palivizumab has been used for over 20 years without concerns for the efficacy or safety of co-administered vaccines, and multiple guidelines recommend the co-administration of palivizumab with routine childhood vaccines [36].

This analysis is limited to safety findings in the year following dosing, a duration corresponding to five nirsevimab elimination half-lives, reflecting a standard approach to safety follow-up in drug development. However, follow-up beyond 1 year is of interest due to the ERD observed with the FI-RSV vaccine [5], where a pathogenic immune response resulted in poorly neutralizing, non-protective antibodies [37,38] and the formation of immune complexes in the lungs [39] that activated complement and enhanced Th2-mediated inflammation. Nirsevimab is a highly neutralizing antibody [40], but with a long half-life, there is a theoretical risk of antibody-dependent enhancement (ADE) of RSV disease when serum concentrations decline to sub-neutralizing levels, particularly in a subsequent RSV season. However, in the predicted conditions of sub-neutralizing concentrations of nirsevimab in MELODY, the incidence and severity of medically attended RSV-associated LRTI were similar between nirsevimab and placebo recipients, giving no indication of ADEs [41]. Moreover, prophylaxis with nirsevimab did not result in a shift in the burden of disease to the second year of life (361 to 511 days post-dosing) [41]. Notably, nirsevimab does not induce sterilizing immunity, and a natural immune response is observed [40].

Although this analysis includes over 3000 nirsevimab recipients, this sample size may be insufficient to detect events with <0.1% incidence. As nirsevimab is implemented in broader populations, real-world experience and pharmacovigilance activities will be instrumental in the continued accumulation of safety data. An ongoing real-world, open-label Phase 3b trial (NCT05437510) is evaluating the efficacy and safety of the approved dose of nirsevimab versus no intervention for the prevention of hospitalizations due to RSV-related LRTI in 8058 healthy infants born ≥29 wGA [42].

This work represents a comprehensive safety analysis of pivotal trials for the first approved prophylactic to protect all infants against RSV lower respiratory tract disease in their first RSV season and children who remain vulnerable to severe RSV disease in their second RSV season with a single dose per season. The safety profile of nirsevimab administered to infants and children before the RSV season was favorable through 360 days post-dose, regardless of gestational age or underlying comorbidities.

## Figures and Tables

**Table 1:** Baseline demographic and clinical characteristics of the nirsevimab, placebo, and palivizumab recipients in pivotal trials (as-treated population).

	Healthy Term and Preterm Infants Born ≥29 wGA Entering Their First RSV Season ^a^		Infants Eligible for Palivizumab Entering Their First RSV Season				Children with CHD/CLD Entering Their Second RSV Season ^b^		
			Preterm Infants Born ≤35 Weeks 0 Days GA without CHD or CLD		Infants with CHD/CLD				
Characteristic	Nirsevimab (*n* = 2570)	Placebo (*n* = 1284)	Nirsevimab (*n* = 406)	Palivizumab (*n* = 206)	Nirsevimab (*n* = 208)	Palivizumab (*n* = 98)	Nirsevimab/Nirsevimab (*n* = 180)	Palivizumab/Nirsevimab (*n* = 40)	Palivizumab/Palivizumab (*n* = 42)
Median age at randomization, months	2.3	2.2	2.9	2.8	4.8	4.3	4.8	4.6	4.0
Median age at start of Season 2, months	–	–	–	–	–	–	16.7	16.4	15.8
Age group at randomization, months, *n* (%)									
≤3.0	1675 (65.2)	828 (64.5)	214 (52.7)	111 (53.9)	59 (28.4)	29 (29.6)	49 (27.2)	10 (25.0)	14 (33.3)
>3.0 to ≤6	716 (27.9)	362 (28.2)	125 (30.8)	59 (28.6)	84 (40.4)	42 (42.9)	76 (42.2)	19 (47.5)	19 (45.2)
>6.0	179 (7.0)	94 (7.3)	67 (16.5)	36 (17.5)	65 (31.3)	27 (27.6)	55 (30.6)	11 (27.5)	9 (21.4)
Neonates	564 (21.9)	291 (22.7)	40 (9.9)	22 (10.7)	6 (2.9)	7 (7.1)	–	–	–
Female sex, *n* (%)	1208 (47.0)	637 (49.6)	201 (49.5)	92 (44.7)	95 (45.7)	39 (39.8)	81 (45.0)	15 (37.5)	15 (35.7)
Race, ^c^ *n* (%)									
American Indian or Alaska Native	92 (3.6)	52 (4.0)	11 (2.7)	5 (2.4)	0	0	0	0	0
Asian	111 (4.3)	56 (4.4)	26 (6.4)	9 (4.4)	10 (4.8)	5 (5.1)	10 (5.6)	3 (7.5)	2 (4.8)
Black or African American	416 (16.2)	178 (13.9)	49 (12.1)	24 (11.7)	10 (4.8)	5 (5.1)	9 (5.0)	2 (5.0)	1 (2.4)
Native Hawaiian/other Pacific Islander	21 (0.8)	11 (0.9)	3 (0.7)	1 (0.5)	1 (0.5)	0	1 (0.6)	0	0
White	1441 (56.2)	740 (57.6)	304 (74.9)	158 (77.1)	177 (85.1)	86 (87.8)	152 (84.4)	35 (87.5)	38 (90.5)
Other	460 (17.9)	236 (18.4)	10 (2.5)	6 (2.9)	7 (3.4)	0	5 (2.8)	0	0
Multiple categories	25 (1.0)	11 (0.9)	3 (0.7)	2 (1.0)	3 (1.4)	2 (2.0)	3 (1.7)	0	1 (2.4)
Ethnicity, *n* (%)									
Hispanic or Latino	793 (30.9)	375 (29.3)	77 (19.0)	35 (17.1)	22 (10.6)	6 (6.1)	19 (10.6)	2 (5.0)	2 (4.8)
Northern Hemisphere, *n* (%)	1883 (73.3)	922 (71.8)	362 (88.9)	185 (88.9)	207 (99.0)	98 (97.0)	178 (98.9)	39 (97.5)	41 (97.6)
Gestational age group, *n* (%)									
<29 weeks ^d^	–	–	48 (11.8)	28 (13.6)	80 (38.5)	40 (40.8)	71 (39.4)	16 (40.0)	16 (38.1)
≥29 to ≤32 weeks	219 (8.5)	115 (9.0)	91 (22.4)	59 (28.6)	37 (17.8)	12 (12.2)	33 (18.3)	2 (5.0)	8 (19.2)
>32 to <35 weeks	346 (13.5)	173 (13.5)	235 (57.9)	112 (54.4)	27 (13.0)	12 (12.2)	25 (13.9)	4 (10.0)	7 (16.7)
≥35 to <37 weeks	246 (9.6)	121 (9.4)	31 ^e^ (7.6)	7 ^e^ (3.4)	14 (6.7)	9 (9.2)	13 (7.2)	4 (10.0)	3 (7.1)
≥37 weeks	1759 (68.4)	875 (68.1)	1 ^f^ (0.2)	0	50 (24.0)	25 (25.5)	38 (21.1)	14 (35.0)	8 (19.0)
Weight on Day 1, kg, median	5.1	5.0	4.3	4.2	5.0	4.8	9.7	9.8	9.9
Weight group on Day 1, *n* (%)									
<2.5 kg ^g^	216 (8.4)	102 (7.9)	46 (11.4)	28 (13.6)	13 (6.3)	2 (2.0)	–	–	–
<5 kg ^g^	1371 (53.3)	676 (52.6)	243 (60.0)	123 (59.7)	101 (48.6)	51 (52.0)	–	–	–
≥5 kg ^h^	1199 (46.7)	608 (47.4)	162 (40.0)	83 (40.3)	107 (51.4)	47 (48.0)	–	–	–
<7 kg ^g^	–	–	–	–	–	–	4 (2.2)	1 (2.5)	1 (2.4)
≥7 kg ^h^	–	–	–	–	–	–	176 (97.8)	39 (97.5)	41 (97.6)
<10 kg ^g^	–	–	–	–	–	–	99 (55.0)	25 (62.5)	23 (54.8)
≥10 kg ^h^	–	–	–	–	–	–	81 (45.0)	15 (3.5)	19 (45.2)

^a^ Includes infants from Phase 2b weighing < 5 kg and the full MELODY enrollment cohort. ^b^ Before the second season, children with CHD/CLD randomized to nirsevimab in the first season received a single IM dose of 200 mg nirsevimab followed by 4× once-monthly IM doses of placebo (nirsevimab/nirsevimab), and those randomized to palivizumab in the first season were re-randomized 1:1 to either a single IM dose of 200 mg nirsevimab followed by 4× once-monthly IM doses of placebo (palivizumab/nirsevimab) or 5× once-monthly IM doses of palivizumab (15 mg/kg per dose) (palivizumab/palivizumab). ^c^ Race was reported by parents or guardians, and each category (except “multiple categories”) comprises infants for whom only that category was selected; “other” comprises infants whose parents or guardians indicated a category other than those listed, and “multiple categories” comprises those for whom more than one category was selected. ^d^ Infants included in the pooled Phase 2b/MELODY cohort were ≥29 weeks’ gestational age. ^e^ Participants were born 35 weeks 0 days to 35 weeks 6 days GA. ^f^ Inclusion criteria protocol deviation recorded. ^g^ These subgroups are not mutually exclusive. ^h^ These subgroups are not mutually exclusive. Abbreviations: CHD, congenital heart disease; CLD, chronic lung disease of prematurity; GA, gestational age; IM, intramuscular; RSV, respiratory syncytial virus; wGA, weeks’ gestational age.

**Table 2:** Most commonly observed Aes (reported in ≥10% of any treatment group during the first RSV season) through 360 days post-dose by preferred term in healthy term and preterm infants born ≥29 wGA. ^a^ Infants with CHD/CLD or preterm infants born ≤35 weeks 0 days GA without CHD/CLD, and children with CHD/CLD entering their second RSV season ^b^.

	Healthy Term and Preterm Infants Born ≥29 wGA ^a^		Infants Eligible for Palivizumab Entering Their First RSV Season				Children with CHD/CLD Entering Their Second RSV Season ^b^		
			Preterm Infants Born ≤35 Weeks 0 Days GA without CHD or CLD		Infants with CHD/CLD				
Preferred Term, *n* (%)	Nirsevimab (*n* = 2570)	Placebo (*n* = 1284)	Nirsevimab (*n* = 406)	Palivizumab (*n* = 206)	Nirsevimab (*n* = 208)	Palivizumab (*n* = 98)	Nirsevimab/ Nirsevimab (*n* = 180)	Palivizumab/ Nirsevimab (*n* = 40)	Palivizumab/ Palivizumab (*n* = 42)
Upper respiratory tract infection	869 (33.8)	417 (32.5)	110 (27.1)	56 (27.2)	39 (18.8)	23 (23.5)	48 (26.7)	8 (20.0)	9 (21.4)
Nasopharyngitis	523 (20.4)	292 (22.7)	36 (8.9)	20 (9.7)	21 (10.1)	19 (19.4)	26 (14.4)	7 (17.5)	9 (21.4)
Pyrexia	348 (13.5)	152 (11.8)	54 (13.3)	33 (16.0)	29 (13.9)	10 (10.2)	23 (12.8)	9 (22.5)	6 (14.3)
Gastroenteritis	284 (11.1)	128 (10.0)	17 (4.2)	14 (6.8)	8 (3.8)	2 (2.0)	14 (7.8)	2 (5.0)	3 (7.1)
Dermatitis diaper	271 (10.5)	126 (9.8)	17 (4.2)	3 (1.5)	11 (5.3)	3 (3.1)	8 (4.4)	0	1 (2.4)
Rhinitis	252 (9.8)	126 (9.8)	48 (11.8)	27 (13.1)	27 (13.0)	13 (13.3)	29 (16.1)	6 (15.0)	6 (14.3)
Constipation	112 (4.4)	55 (4.3)	16 (3.9)	10 (4.9)	21 (10.1)	10 (10.2)	5 (2.8)	2 (5.0)	2 (4.8)

Participants with multiple events in the same preferred term were counted once in each of those preferred terms. Participants with events in more than one preferred term were counted once in each of those preferred terms. ^a^ Includes infants from Phase 2b weighing < 5 kg and the full MELODY enrollment cohort. ^b^ Before the second season, children with CHD/CLD randomized to nirsevimab in the first season received a single IM dose of 200 mg nirsevimab followed by 4× once-monthly IM doses of placebo (nirsevimab/nirsevimab), and those randomized to palivizumab in the first season were re-randomized 1:1 to either a single IM dose of 200 mg nirsevimab followed by 4× once-monthly IM doses of placebo (palivizumab/nirsevimab) or 5× once-monthly IM doses of palivizumab (15 mg/kg per dose) (palivizumab/palivizumab). Abbreviations: AE, adverse event; CHD, congenital heart disease; CLD, congenital lung disease; GA, gestational age; IM, intramuscular; RSV, respiratory syncytial virus; wGA, weeks’ gestational age.

**Table 3:** Most commonly observed serious AEs (reported in ≥0.5% and ≥2 participants in any treatment group during the first RSV season) in healthy term and preterm infants born ≥29 wGA, infants with CHD/CLD, preterm infants born ≤35 weeks 0 Days GA without CHD/CLD, and children with CHD/CLD entering their second RSV season.

	Healthy Term and Preterm Infants Born ≥29 wGA ^a^		Infants Eligible for Palivizumab Entering Their First RSV Season				Children with CHD/CLD Entering Their Second RSV Season ^b^		
			Preterm Infants Born ≤35 Weeks 0 Days GA without CHD or CLD		Infants with CHD/CLD				
Preferred Term, *n* (%)	Nirsevimab (*n* = 2570)	Placebo (*n* = 1284)	Nirsevimab (*n* = 406)	Palivizumab (*n* = 206)	Nirsevimab (*n* = 208)	Palivizumab (*n* = 98)	Nirsevimab/ Nirsevimab (*n* = 180)	Palivizumab/ Nirsevimab (*n* = 40)	Palivizumab/ Palivizumab (*n* = 42)
Bronchiolitis	37 (1.4)	33 (2.6)	4 (1.0)	0	7 (3.4)	4 (4.1)	1 (0.6)	0	0
Pneumonia	21 (0.8)	12 (0.9)	2 (0.5)	0	3 (1.4)	1 (1.0)	2 (1.1)	2 (5.0)	0
Gastroenteritis	19 (0.7)	7 (0.5)	0	1 (0.5)	6 (2.9)	0	3 (1.7)	1 (2.5)	1 (2.4)
LRTI	16 (0.6)	10 (0.8)	0	0	1 (0.5)	2 (2.0)	2 (1.1)	0	0
Bronchitis	13 (0.5)	13 (1.0)	3 (0.7)	1 (0.5)	2 (1.0)	1 (1.0)	0	0	0
Urinary tract infection	7 (0.3)	8 (0.6)	1 (0.2)	0	1 (0.5)	1 (1.0)	0	0	0
RSV bronchiolitis	6 (0.2)	12 (0.9)	0	1 (0.5)	4 (1.9)	1 (1.0)	0	0	0
Upper respiratory tract infection	6 (0.2)	5 (0.4)	0	2 (1.0)	1 (0.5)	1 (1.0)	1 (0.6)	0	0
Viral upper respiratory tract infection	5 (0.2)	0	0	0	3 (1.4)	1 (1.0)	1 (0.6)	0	0
COVID-19	3 (0.1)	2 (0.2)	3 (0.7)	1 (0.5)	0	0	2 (1.1)	0	0
Inguinal hernia	1 (<0.1)	6 (0.5)	1 (0.2)	1 (0.5)	0	0	0	0	0
Cardiac failure	1 (<0.1)	0	0	0	1 (0.5)	2 (2.0)	0	0	0
Failure to thrive	1 (<0.1)	0	0	0	2 (1.0)	0	1 (0.6)	0	0
Bradycardia	0	0	1 (0.2)	2 (1.0)	0	0	0	0	0
Feeding intolerance	0	0	0	0	2 (1.0)	0	0	0	0
Pleural effusion	0	0	0	0	0	0	2 (1.1)	0	0

Participants with multiple events in the same preferred term were counted once in each of those preferred terms. Participants with events in more than one preferred term were counted once in each of those preferred terms. Serious AEs were defined as death, life-threatening, requiring inpatient hospitalization, prolongation of existing hospitalization, persistent or significant disability/incapacity, important medical event, or congenital anomaly/birth defect. ^a^ Includes infants from Phase 2b weighing < 5 kg and the full MELODY enrollment cohort. ^b^ Before the second season, children with CHD/CLD randomized to nirsevimab in the first season received a single IM dose of 200 mg nirsevimab followed by 4× once-monthly IM doses of placebo (nirsevimab/nirsevimab), and those randomized to palivizumab in the first season were re-randomized 1:1 to either a single IM dose of 200 mg nirsevimab followed by 4× once-monthly IM doses of placebo (palivizumab/nirsevimab) or 5× once-monthly IM doses of palivizumab (15 mg/kg per dose) (palivizumab/palivizumab). Abbreviations: AE, adverse event; CHD, congenital heart disease; CLD, congenital lung disease; COVID-19, coronavirus disease 2019; GA, gestational age; IM, intramuscular; LRTI, lower respiratory tract infection; RSV, respiratory syncytial virus; wGA, weeks’ gestational age.

## Data Availability

Data underlying the findings described in this manuscript may be obtained in accordance with AstraZeneca’s data sharing policy described at https://www.astrazenecaclinicaltrials.com/our-transparency-commitments/ (accessed on 18 April 2024). Data for studies directly listed on Vivli can be requested through Vivli at www.vivli.org. Data for studies not listed on Vivli can be requested through Vivli at https://vivli.org/members/enquiries-about-studies-not-listed-on-the-vivli-platform/. The AstraZeneca Vivli member page is also available outlining further details: https://vivli.org/ourmember/astrazeneca/ (accessed on 18 April 2024).

## Supplementary Materials

The following supporting information can be downloaded at: https://www.mdpi.com/article/10.3390/pathogens13060503/s1, Section S1: Study Designs; Section S2: Subgroups of Interest; Section S3: Independent Ethics Committees/Institutional Review Boards consulted; Figure S1: Participant disposition (CONSORT diagram): healthy preterm and term infants in the Phase 2b and MELODY trials; Figure S2: Participant disposition (CONSORT diagram): infants with CHD/CLD and preterm infants born ≤35 weeks 0 days GA without CHD/CLD in the MEDLEY trial; Figure S3: Summary of AEs through 360 days post-dose in healthy term and preterm infants born ≥29 wGA who were (A) neonates or (B) weighed <2.5 kg at dosing and infants at higher risk of severe disease eligible for palivizumab who were (C) born <29 wGA, (D) with CHD, or (E) with CLD; Figure S4: Summary of AEs within 30 days of dosing in (A) preterm infants born ≤35 weeks 0 days GA without CHD/CLD or (B) infants with CHD/CLD entering their first RSV season and (C) children with CHD/CLD entering their second RSV season; Figure S5: Summary of AEs in children with CHD/CLD entering their second RSV season through (A) 360 days and (B) 30 days post first dose in the second season; Figure S6: Nirsevimab serum AUC0–365 (A and B) and Cmax (C and D) in participants with ≥Grade 3 AEs or serious AEs (A and C) and AESI (B and D); Table S1: Treatment Exposure and Follow-Up; Table S2: AESI Among Nirsevimab Recipients Across Pivotal Trials Through 360 Days Post-Dose; Table S3: Most Common AEs (≥1% in Any Group) by Preferred Term within 14 Days Post-Dosing with or without Co-Administered Vaccine in Healthy Term and Preterm Infants Born ≥29 wGA; Table S4: Treatment-Related AEs by System Organ Class and Preferred Term in Infants Entering Their First RSV Season Through 360 Days Post-Dose; Table S5: Nirsevimab Recipients with Post-Baseline Antidrug Antibodies Through 360 Days Post-Dose; Table S6: AEs by Post-Baseline Antidrug Antibody Status Through 360 Days Post-Dose; Table S7: AEs by Post-Baseline Antidrug Antibody Status in Children with CHD/CLD Entering Their Second RSV Season Through 360 Days Post-Dose; Table S8: Selected AEs Typically Solicited in Vaccine Studies in Infants within 7 Days Post-Dose in Healthy Term and Preterm Infants Born ≥29 wGA; Table S9: Serious AEs by System Organ Class and Preferred Term in Children with CHD/CLD Entering Their Second RSV Season Through 360 Days Post-Dose; Table S10: Treatment-Emergent AEs of Grade 3 or Greater Severity by System Organ Class and Preferred Term in Children with CHD/CLD Entering Their Second RSV Season Through 360 Days Post-Dose; Table S11: Fatal Events in Healthy Term and Preterm Infants ≥29 wGA Through 360 Days Post-Dose; Table S12: Fatal Events in Infants At Higher Risk of Severe RSV Disease Through 360 Days Post-Dose.
