# Supplementary material for: Comprehensive Summary of Safety Data on Nirsevimab in Infants and Children from All Pivotal Randomized Clinical Trials
Source: Pathogens. 2024 Jun 13;13(6):503. doi: 10.3390/pathogens13060503 (PMC11206492; doi:10.3390/pathogens13060503)
Supplement: Supplementary file 1 [file pathogens-13-00503-s001.zip › pathogens-2997340-supplementary.pdf]

# Comprehensive Summary of Safety Data on Nirsevimab in Infants and Children from All Pivotal Randomized Clinical Trials

Vaishali S. Mankad, Amanda Leach, Yue Chang, Ulrika Wählby Hamrén,

Alexandre Kiazand, Robert J. Kubiak, Therese Takas, Tonya Villafana, Manish Shroff

**SUPPLEMENTARY INFORMATION**

**Section S1: Study Designs**

In MEDLEY Season 1, treatment-emergent adverse events (AEs) were any AE that started on/after the Season 1 Day 1 dose (start of Season 1) and prior to the end of Season 1, Day 361 (end of Season 1) or the last Day prior to Season 2, Day 1 (start of Season 2). For MEDLEY Season 2, treatment-emergent AEs were any AEs that started on/after the date of the Season 2, Day 1 dose (start of Season 2) and prior to the end of Season 2, Day 361 (end of Season 2). Safety analysis in MEDLEY also included AEs within 30 days post first dose in each season to enable assessment of AEs following the active nirsevimab dose compared with one active palivizumab dose. AEs leading to discontinuation of treatment were evaluated only in MEDLEY, the only study entailing multiple doses of treatment per season whereas AEs leading to discontinuation from the study were captured for all studies.

**Section S2: Subgroups of Interest**

Clinically relevant subgroups evaluated in this analysis included neonates (defined as those <28 days age at randomization) and infants weighing <2.5 kg on Day 1 (representing those who had relatively higher nirsevimab exposures of approximately 20–30 mg/kg in their first season) among the healthy preterm and term infants included from the Phase 2b and MELODY trials. Among children at higher risk for severe RSV disease in the MEDLEY trial, subgroups evaluated included infants born <29 weeks gestational age (wGA) (irrespective of congenital heart disease [CHD]/chronic lung disease [CLD] status), infants with CHD, and infants with CLD.

**Section S3: Independent Ethics Committees/Institutional Review Boards consulted**

| Country | Name/Address of IRB/IEC | Site number |
|---|---|---|
| **MEDLEY (NCT03959488)** | | |
| Austria | Ethikkommission der Medizinischen Universität Graz, Auenbruggerpl. 2, 8036 Graz, Austria | 2004190 |
| Belgium | O.L.V. Ziekenhuis,Moorselbaan, 164, Aalst, 9300 | 2004512, 2005091 |
| Bulgaria | Ethics Committee for Clinical Trials, 8, Damyan Gruev Str., Sofia,1303 | 2004194, 2004483, 2004484, 2004511, 2004531, 2004559, 2004580, 2004596, 2004770, 2005099 |
| Canada | UBC C&W Research Ethics Board A2-141A, 950 West 28th Avenue Vancouver, BC V5Z 4H4<br>Health Research Ethics Board of Alberta, 10104 103 Ave NW #1500, Edmonton, AB T5J 0H8 | 2004506<br>2005229 |
| Czech Republic | Multicentricka eticka komise, IKEM a TN, Videnska 800, Praha,140 59 | 2004564 |
| Estonia | Research Ethics Committee of the National Institute for Health Development,Hiiu 42, Tallinn, 11619 | 2004062, 2004591 |
| Finland | Varsinais-Suomen sairaanhoitopiiri Eettinen toimikunta, Kiinamyllynkatu 4-8,PL 52 Turku, 20520 | 2004592 |
| France | Comité de Protection des Personnes Sud Ouest et Outre Mer III, 146 Rue Léo Saignat, 33000, Bordeaux, France | 2004064, 2004196, 2004197, 2004515, 2004561, 2004565, 2004579, 2004586, 2005096 |
| Germany | Ethik-Kommission an der Medizinischen Fakultaet der Universitaet Leipzig, Stephanstraße 9A.1, EG 04103 Leipzig | 2004207, 2004211, 2004597 |
| Hungary | Egeszsegugyi Tudomanyos Tanacs Klinikai Farmakologiai Etikai Bizottsaga, 25 Alkotmány u., Budapest, H-1054, Hungary | 2004066, 2004067, 2004491, 2004566,<br>2005078 |
| Italy | Comitato Etico per la Sperimentazione Clinica delle Provincie di Verona e Rovigo, P.le Stefani, 1, Verona,37126 | 2004069, 2004557 |
| Japan | Fukuoka Children's Hospital IRB, 5 Chome-1-1 Kashiiteriha, Higashi Ward, Fukuoka, 813-0017, Japan | 2004472 |

| Country | Name/Address of IRB/IEC | Site number |
|---|---|---|
| | Fukui-ken Saiseikai Hospital Institutional Review Board, 7-1 Funabashi, Wadanaka-cho, Fukui-shi, Fukui-Ken " | 2004539 |
| | Japanese Red cross Maebashi Hospital IRB, 138-Asakuramachi, Maebashi-Shi Gunma | 2004599 |
| | Saitama City Hospital IRB, 2460 Mimuro, Midori Ward, Saitama, 336-8522, Japan | 2004698 |
| | JCHO Kyushu Hospital IRB, 1 Chome-8-番１号 Kishinoura, Yahatanishi Ward, Kitakyushu, Fukuoka 806-8501, Japan | 2004773 |
| | National Center for Child Health and Development IRB, 2-chōme-10-1 Ōkura, Setagaya City, Tokyo 157-0074, Japan | 2004774 |
| Latvia | Ethics Committee for Clinical Trials of Medicinal Products, Aizkraukles street 21 - 113, Riga, LV1006 | 2004246, 2004516, 2004519 |
| Lithuania | Lithuanian Bioethics Committee, Algirdo g. 31, Vilnius, LT-03219 | 2004247, 2004248 |
| Mexico | Comité de Ética en Investigacion del Hospital Infantil de Mexico Federico Gomez, Calle Doctor Márquez 162 Delegación, Doctores, Cuauhtémoc, 06720 Ciudad de México, CDMX, Mexico | 2004529 |
| | Comite de Etica en Investigacion de Investigacion Biomedica para el Desarrollo de Farmaco Insurgentes, Sur #662 PB, Delegación Benito Juárez Del Valle Centro | 2004555 |
| Poland | Komisja Bioetyczna przy Okregowej Izbie Lekarskiej w Rzeszowie, ul. Jana Dekerta 2, Rzeszów,35-030 | 2004133, 2004137, 2004141, 2004467, 2004583 |
| Republic of Korea | Samsung Medical Center Institutional Review Board, (06351) 81 Irwon-Ro Gangnamgu, Seoul, Korea | 2004776 |
| | IRB of Ajou University Hospital, San 5, Woncheon-dong, Yeongtong-gu, Suwon 443-721, Republic of Korea | 2004777 |
| | IRB of Korea University Ansan Hospital, 123 Jeokgeum-ro, Danwon-gu, Ansan-si, Gyeonggi-do, South Korea | 2004803 |
| Russia | Ethical Council at the MoH of RF, 3 Rakhmanovsky Pereulok, Moscow,127994 | 2004074, 2004143, 2004144, 2004521, 2004551, 2004568 |
| | LEC at SBHI Novosibirsk City, Clinical Perinatal Center. Ulitsa Adriyena Lezhena, 32, Novosibirsk, Novosibirsk Oblast, Russia, 630089 | 2005178 |
| South Africa | University of Witwatersrand, Human Research Ethics Committee, 1 Jan Smuts Avenue, Braamfontein 2000, Johannesburg, South Africa | 2004076, 2004078, 2004147 |
| | Pharma Ethics, 123 Amcor Road Lyttlelon Manor, South Africa 0157 | 2004077, 2004079 |
| | 1 Military Hospital Human Research Ethics Committee, Department of Neurology, Private bag X 1026 Thaba Tswane 0143 | 2004080 |
| | Stellenbosch University Human Research Ethics Committee, Stellenbosch University, Private Bag X1, Matieland, 7602, Stellenbosch, South Africa | 2004477 |
| Spain | Hospital Universitario Clinico San Carlos, Puerta G - Planta 4ª Norte,C/ Profesor Martin Lagos, s/n Madrid, 28040 | 2004082, 2004083, 2004084, 2004150, 2004493, 2004500, 2004545, 2004553, 2004587, 2005180, 2005181 |

| Country | Name/Address of IRB/IEC | Site number |
|---|---|---|
| Sweden | Etikprövningsmyndigheten, Box 2110,SE-750 02 Uppsala,SE-750 02 | 2004087 |
| Turkey | Ege University Ethics Committee, Ege Universitesi Tip Fakultesi, Klinik Arastirmalar Etik Kurulu Izmir, 35100 | 2004152, 2004154, 2004156 |
| Ukraine | Dept of health of Chernivtsi city council, Communal Medical Institution City Clinical Childrens' Hospital, 4 Bukovynska St, Chernivtsi, 58001 | 2004089 |
| | Vinnytsia regional Children's Clinical Hospital, 108 Khmelnytske shose st, Vinnytsia, 21000. Medical Ethics Commission | 2004157 |
| | Ministy of Health of Ukraine, Communal Non-Commercial enterprise Saint Zinaida Children's Clinical Hospital of Sumy City Council, 28 Troiiska st, Sumy, 40022 | 2004159 |
| | Communal Non-Commercial enterprise of Kharkiv Regional Council regional Children's clinical hospital, 5 Ozeryanska st Kharkiv, 61093 | 2004517 |
| | Odesa Regional State administration, department of health, communal enterprise, Odesa regional Children's clinical hospital, 3 Ac Vorobiov st, Odes-31, 65031 | 2004522 |
| | Ethics Commission at Communal Institution Dnipro City Children's Clinical Hospital No 5 of Dnipro City Council, 5 ivana Akinfiieva, st, Dnipro 49027 Ukraine | 2004570 |
| | LEC Reg Dniprop Children Clin Hosp, Karavajeva St, 68, Dnipro, Dnipropetrovsk Oblast, Ukraine, 49000 | 2005230 |
| | LEC Ivano-Frankivsk Regional Children Clinical Hospital, Viacheslava Chornovola St, 44, Ivano-Frankivsk, Ivano-Frankivsk Oblast, Ukraine, 76000 | 2005231 |
| United Kingdom | NRES Committee South Central - Berkshire,South West REC Centre,Level 3, Block B, Bristol, BS1 2NT | 2004482, 2004594, 2005077 |
| United States of America | WCG IRB, 212 Carnegie Center, Suite 301, Princeton, NJ 08540, USA | 2004094, 2004161, 2004176, 2004584, 2004164, 2004168, 2004585, 2004169, 2004171, 2004172, 2004461, 2004524, 2004547, 2004549, 2004571, 2004589, 2004606, 2004735, 2005100, 2005182, 2005232 |
| | Institutional Review Board, Ann & Robert H. Lurie Children's Hospital of Chicago, 25 East Chicago Avenue, Chicago, Illinois" | 2004177 |
| | Cincinnati Children’s Hospital Institutional Review Board, 3333 Burnet Avenue \| MLC 7040 \| Cincinnati, OH 45229 | 2004179 |
| | Institutional Review Boards, University of Louisville 2301 S 3rd St, Louisville, KY 40292, United States | 2004463 |
| | Duke University Health System, Institutional Review Board, 2301 Erwin Rd, Durham, NC 27710, United States | 2004603 |
| | Childrens Hospital of Los Angeles-Committee on Clinical Investigations IRB, 4661 Sunset Blvd, Los Angeles, CA 90027, United States | 2004705 |
| | East Carolina University, University and Medical Center Institutional Review Board | 2005208 |

| Country | Name/Address of IRB/IEC | Site number |
|---|---|---|
| **Phase 2b (NCT02878330)** | | |
| Argentina | Comité de Ética en Investigación Científica. Hospital Pediátrico Dr. Humberto Notti Bandera de Los Andes 2603 Villa Nueva Guaymallén Mendoza | 2002905 |
| | Comité Hospitalario de Etica Necochea 675 Bahia Blanca Buenos Aires | 2002909 |
| Australia | Monash Health Human Research Ethics Committee (RGO) Level 2, I Block Clayton Victoria | 2002910 |
| | Royal Children's Health Services Human Research Ethics Committee (RGO) 50 Flemington Road Parkville Victoria | 2002911 |
| | Princess Margaret Hospital for Children Ethics Committee Princess Margaret Hospital Entrance No 6, Hamilton Street Subiaco Western Australia | 2002912 |
| Belgium | Comité d'Ethique du CHU Ambroise Paré Boulevard Kennedy 2 Mons | 2003274 |
| Brazil | CEP Investiga - Instituto de Pesquisas Avenida Romeu Tortima, 739 - Cidade Universitária Campinas Sao Paulo | 2002934 |
| | CEP da Universidade Federal de Minas Gerais Avenida Presidente Antonio Carlos 6627 Unidade Administrativa II Belo Horizonte Minas Gerais | 2002935 |
| | CEP da Faculdade de Ciências Médicas e da Saúde de Juiz de Fora SUPREMA/MG Alameda Salvaterra, 200 Bairro Salvaterra Juiz de Fora Minas Gerais | 2002939 |
| | CEP da Faculdade de Medicina de Botucatu - UNESP/SP Distrito de Rubião Junior Botucatu Sao Paulo | 2003060 |
| | Comitê de Ética em Pesquisa em Seres Humanos do Instituto de Medicina Integral Professor Fernando F Rua dos Coelhos, 300 -Boa Vista Recife Pernambuco | 2002940 |
| | CEP da Universidade Luterana do Brasil -ULBRA Farroupilha, 8001 - Prédio 14 - Sala 224 Bairro São José Canoas Rio Grande do Sul | 2002941 |
| | Comitê de Ética em Pesquisa em Seres Humanos do Hospital Pequeno Príncipe Rua Desembargador Motta, 1070 6º andar, sala do NUPE Curitiba Paraná | 2002943 |
| | CEP da Universidade de Passo Fundo/RS Universidade de Passo Fundo - BR 285, Bairro São José Passo Fundo Rio Grande do Sul | 2002944 |
| Canada | McGill University Health Center-Research Ethics Board 2155 Guy Street 2nd Floor, Room 231 Montreal Quebec | 2003277, 2003280 |
| Chile | Comite Etico Cientifico del Servicio de Salud Metropolitano Sur Santa Rosa 3453, Piso 1 San Miguel Santiago | 2002998, 2002999 |
| | Comité Etico Científico Servicio de Salud Valdivia Maipú 550, oficina 307 Valdivia | 2002953 |
| | Comité Ético Científico Servicio de Salud Metropolitano Central Victoria Subercaseaux 381, piso 4 Santiago | 2002956 |
| | Comité Ético-Científico Servicio de Salud Metropolitano Sur Oriente Av Concha y Toro 3459 Puente Alto Santiago | 2002954 |
| | Comité Ético-Científico Servicio de Salud Viña del Mar-Quillota Calle Limache #1307 Esquina Peñablanca 2º Piso Viña del Mar | 2003257 |

| Country | Name/Address of IRB/IEC | Site number |
|---|---|---|
| | Comité de Ética de Investigación en Seres Humanos Av. Independencia 1027, Independencia Santiago | 2002955 |
| | Comité de Ética de la Investigación Servicio de Salud Metropolitano Norte San José #1053 Independencia Santiago | 2002955 |
| Czech Republic | Eticka komise Ustav pro peci o matku a dite Podolske nabrezi 157/36 Praha 4 - Podoli | 2002926 |
| | Eticka komise Nemocnice Havlickuv Brod Husova 2624 Havlickuv Brod | 2002927 |
| | Eticka komise IKEM a FTNsP Videnska 800 Praha 4 - Krc | 2002924 |
| Italy | CESC della Provincia di Padova Presso Azienda Ospedaliera di Padova_Via Giustiniani 1 Padova Padova | 2003034 |
| | Comitato Etico per la Sperimentazione Clinica delle Provincie di Verona e Rovigo P.le Stefani, 1 Verona Verona | 2003000 |
| | Azienda Ospedaliera Città della Salute e della Scienza di Torino Corso Bramante 88/90. Torino Torino | 2003065 |
| | Comitato Etico Regionale della Liguria Largo Rosanna Benzi 10 Farmacia Ospedaliera Genova Genova | 2003035 |
| South Africa | Pharma Ethics 123 Amcor Road Lyttelton Manor Centurion Pretoria Gauteng | 2002923, 2002919, 2002921, 2002938 |
| | Wits Health Consortium 31 Princess of Wales Terrace Parktown Johannesburg Gauteng | 2002918, 2002937 |
| | University of Stellenbosch Ethics Committee Faculty of Health Sciences Francie van Zijl Drive Tygerberg Cape Town Western Cape | 2002920 |
| | University of Cape Town HREC Faculty of Health Sciences Research EC E52-24 Old Main Building Groote Schuur Hospital, Observatory Cape Town Western Cape | 2002922, 2002946 |
| Spain | CEIC de Galicia C/ San Lázaro, s/n Secretaria Xeral. Conselleria de SanidadeDirección Santiago de Compostela La Coruña | 2002947, 2002948, 2002950, 2002951 |
| United Kingdom | R&D University Hospital Southampton NHS Foundation Trust Tremona Road, Level E, Laboratory & Pathology Block, SCBR - MP 138 Southampton Hampshire | 2002967 |
| | R&D - Brighton and Sussex University Hospitals Royal Sussex County Hospital Level 5 Thomas Kemp Tower Eastern Road Brighton East Sussex | 2003320 |
| | R&D - Alder Hey Children's NHS Foundation Trust Eaton Road Liverpool Merseyside | 2003319 |
| | R&D South West London and St George's Mental Health NHS Trust Department of Mental Health, St George's, University of London, 6th Floor, Hunter Wing, Cranmer Terrace London Greater London | 2002968 |
| | R&D - CRN Thames Valley and South Midlands 1st Floor, Manor House The John Radcliffe Hospital, Headley Way Headington Oxford Oxfordshire | 2002969 |
| | R&D University Hospitals Bristol NHS Foundation Trust Education & Research Centre Level 3 Upper Maudlin Street Bristol Avon | 2002966 |
| United States | MetroHealth Medical Center IRB 2500 MetroHealth Dr. Rammelkamp Bldg. Room 103 Cleveland Ohio | 2003359 |

| Country | Name/Address of IRB/IEC | Site number |
|---|---|---|
| | Copernicus Group IRB 5000 CentreGreen Way Suite 200 Cary North Carolina | 2003395, 2003091, 2002970, 2003007, 2003356, 2003405, 2003355, 2003354, 2003004, 2003353, 2002971, 2003124, 2003350, 2003394, 2003349, 2003092, 2003348, 2003078, 2002974, 2003036, 2003346, 2003340, 2003441, 2003167, 2003338, 2003337, 2003442, 2003068, 2003038, 2003342, 2003335, 2003399, 2003086, 2003444, 2003400, 2003332, 2002976, 2003402, 2003347, 2003403, 2003125, 2003329, 2003336, 2003079, 2003401, 2003407 |
| | Medical University of South Carolina IRB 19 Hagood Avenue 6th floor, Suite 601 Charleston South Carolina | 2003358 |
| | UTHSC IRB Office 910 Madison Suite 600 Memphis Tennessee | 2003352 |
| | Memorial Health Services Research Council 2801 Atlantic Avenue Attn Research Administration Long Beach California | 2003118 |
| | SUNY IRB 750 East Adams Street CWB 218G Syracuse New York | 2002972 |
| | WIRB 1019 39th Avenue SE Suite 120 Puyallup Washington | 2002973, 2003061, 2003069, 2003093, 2003331, 2003447 |
| | Sharp Healthcare IRB 7930 Frost St Suite 300 San Diego California | 2003343 |
| | Winthrop-University Hospital IRB 222 Station Plaza North Suite 521 Mineola New York | 2003341 |
| | Marshall University Office of Research Integrity One John Marshall Drive Huntington West Virginia | 2003339 |
| | Ann & Robert H. Lurie Children’s Hospital of Chicago Institutional Review Board 225 E. Chicago Avenue Box 59 Chicago Illinois | 2003011 |
| | Chesapeake IRB 7063 Columbia Gateway Drive Suite 110 Columbia Maryland | 2003334 |
| | Cincinnati Children's Hospital Medical Center IRB 3333 Burnet Ave. MLC 5020 Cincinnati Ohio | 2003067 |
| | Childrens Hospital of Los Angeles-Committee on Clinical Investigations IRB 4650 Sunset Blvd Mail Stop #23 Dr. Andreas Reiff Los Angeles California | 2003333 |
| | University of Texas at San Antonio IRB One UTSA Circle MS 4.01.82 San Antonio Texas | 2003005 |
| | Chesapeake IRB 6940 Columbia Gateway Dr #110 Columbia Maryland | 2002975 |
| | Arnold Palmer Medical Center Institutional Review Board 1401 Kuhl Avenue MP #21 Research Department Orlando Florida | 2003330 |
| | University of Nebraska Medical Center IRB 987830 Nebaska Medical Center Omaha Nebraska | 2003328 |
| | Creighton University IRB 2500 California Plaza IRB-Biomedical Omaha Nebraska | 2003009 |
| | Oklahoma University Health Sciences Center 1105 North Stonewall Avenue Oklahoma City Oklahoma | 2003448 |
| | Connecticut Children's Medical Center IRB 282 Washington Street. Suite 2 K. Hartford Connecticut | 2003406 |

| Country | Name/Address of IRB/IEC | Site number |
|---|---|---|
| **MELODY (NCT03979313)** | | |
| Argentina | Independent Ethics Committee for Clinical Pharmacology Trials, Drug and Pharmacology Studies Foundation | 2004648, 2004132 |
| Australia | Child and Adolescent Health Service (HREC), Office 5E, Perth Children's Hospital,15 Hospital Avenue Nedlands,6009 | 2004373, 2004327 |
| Austria | Ethikkommission der Medizinischen Universität Graz, Auenbruggerplatz 2, Graz,8036 | 2004249, 2004298 |
| Belgium | O.L.V. Ziekenhuis, Moorselbaan 164, Aalst,9300 | 2004629, 2004231, 2004270, 2004299, 2004320, 2004398, 2004320 |
| Bulgaria | Ethics Committee for Clinical Trials,8, Damyan Gruev Str.,, Sofia,1303 | 2004303, 2004234, 2004325, 2004339, 2004343, 2004639, 2004324, 2004401, 2004399 |
| Canada | Conjoint Health Research Ethics Board Research Services Office 2500 University Drive, NW Calgary AB T2N 1N4 | 2004667 |
| | MUHC Centre for Applied Ethics 5100, boul. de Maisonneuve Ouest, 5th floor, Office 576 Montréal, Québec, H4A 3T2 | 2004365, 2004351 |
| | UBC C&W Research Ethics Board A2-141A, 950 West 28th Avenue Vancouver, BC V5Z 4H4 | 2004623 |
| Chile | Servicio De Salud Metropolitano Sur Oriente Comite Etico- Cientifico, Av. Concha y Toro 3459 – Paradero 30, Vic. Mackenna | 2004043 |
| | UNIVERSIDAD DE CHILE [University of Chile] – FACULTAD DE MEDICINA HUMAN RESEARCH ETHICS COMMITTEE, Av. Libertador Bernardo O'Higgins 1058, Santiago de Chile | 2004098 |
| | Ministerio de Salud Servicio de Salud Valdivia Scientific Ethics Committee, V. Pérez Rosales 560 - Edificio Prales - Oficina 307 - Piso 3 | 2004296 |
| | Servicio de Salud Metropolitano Norte Research Ethics Committee, 272,Calle Maruri 8380000 Independencia Metropolitana de Santiago | 2004300 |
| Colombia | Universidad Pontificia Bolivariana, Calle 78 B No. 72 A 109 | 2004233 |
| | CORPORACIÓN CIENTÍFICA PEDIÁTRICA, BIOMEDICAL RESEARCH ETHICS COMMITTEE, Calle 5 B5 No. 37 bis - 28 | 2004295 |
| | UNIVERSIDAD CES, Calle 10A No. 22 - 04 El Poblado | 2004304 |
| | COMITÉ DE ÉTICA EN INVESTIGACIÓN VIT, Calle 24 N° 3-02 este | 2004372 |
| | Research Ethics Committee of the Health Sciences Department of the Universidad del Norte, Apartados Aéreos 1569 - 51820, Km. 5 vía Puerto Colombia | 2004400 |
| | State Social Enterprise HOSPITAL MENTAL DE ANTIOQUIA [Antioquia Psychiatric Hospital], Calle 38 55- 310 Bello-Colombia | 2004669 |
| | Human Research Ethics Committee Fundación Hospital Infantil Universitario de San José, Carrera 52 No. 67 A-71 PBX: 4377540 | 2004681 |
| Czech Republic | Multicentricka eticka komise IKEM a TN,Videnska 800, Praha,140 58 | 2004271 |
| | Multicentricka eticka komise IKEM a TN,Videnska 800, Praha,140 59 | 2004272, 2004044 |

| Country | Name/Address of IRB/IEC | Site number |
|---|---|---|
| Estonia | Research Ethics Committee of the National Institute for Health Development,Hiiu 42, Tallinn,11619 | 2004273, 2004045, 2004046, 2004099, 2004047, 2004274 |
| Finland | Varsinais-Suomen sairaanhoitopiiri Eettinen toimikunta ,Kiinamyllynkatu 4-8,PL 52 Turku,20520 Hospital District of Southwest Finland Joint Municipal Authority Ethics Committee, Turku University Hospital, T-Hospital, 6th Floor, Board meeting room A 607 | 2004742,2 2004741, 2004313, 2004743, 2004611, 2004312, 2004644 2004887, 2004896 |
| France | Comité de Protection des Personnes Ile de France VIII, Hôpital Ambroise Paré, 9 avenue Charles de Gaulle Boulogne Billancourt,92100 | 2004654, 2004100, 2004232, 2004374, 2004378, 2004646, 2004653, 2004676, 2005602 |
| Germany | Ethik-Kommission an der Medizinischen Fakultaet der Universitaet Leipzig, Kaethe-Kollwitz-Strasse 82,Haus: Karl-Sudhoff-Institut Leipzig,04109 | 2004185 |
| | Landesärztekammer Baden-Württemberg Ethik-Kommission Liebknechtstr. 33 70565 Stuttgart | 2004178 |
| | Ethik-Kommission der Bayerischen Landesärztekammer Mühlbaurstr.16 D-81677 München | 2004182 |
| | Ethikkommission der Landesärztekammer Rheinland-Pfalz Deutschhausplatz 3 55116 Mainz | 2004359 |
| Israel | Soroka University Medical Center, Itzhak Rager Blv. Beer Sheva 8458900 | 2004626 |
| | Laniado Hospital, 16 deuteronomy haim st., kiryat sanz netanya, 42150 | 2004672 |
| Italy | Comitato Etico per la Sperimentazione Clinica delle Provincie di Verona e Rovigo,P.le Stefani, 1, Verona,37126 | 2004103 |
| | COMITATO ETICO DELLA FONDAZIONE POLICLINICO UNIVERSITARIO AGOSTINO GEMELLI IRCCS UNIVERSITÀ CATTOLICA DEL SACRO CUORE | 2004396 |
| Japan | Japanese Red cross Maebashi Hospital IRB 138-Asakuramachi, Maebashi-Shi Gunma | 2004632 |
| | Yokosuka Kyosai Hospital IRB, 1-16 Yonegahamadori, Yokosuka Kanagawa | 2004660 |
| | Jimbo Orthopedic Surgery Institutional Review Board 5-38-41, Honcho Koganei-shi, Tokyo | 2004668 |
| | NHO Okayama Medical Center IRB Kita-ku Tamasu 1711-1, Okayama-shi, Okayama-Ken, Japan | 2004670 |
| | Kawasaki Municipal Hospital Institutional Review Board 12-1, Shinkawa-dori, Kawasaki-ku, Kawasaki-shi, Kanagawa | 2004671 |
| | Fukuyama City Hospital Institutional Review Board 5-23-1 Zao-cho, Fukuyama-shi, Hiroshima | 2004687 |
| | KKR Sapporo Medical Center IRB 6-3-40 Hiragishi 1-jo Toyohira-ku, Sapporo-shi, Hokkaido | 2004688 |
| | Fukui-ken Saiseikai Hospital Institutional Review Board 7-1 Funabashi, Wadanaka-cho, Fukui-shi, Fukui-Ken | 2005029 |
| | Institutional Review Board of Okayama City General Medical Center Okayama City Hospital 3-20-1 Kitanagaseomotemachi, Kita-ku, Okayama-shi, Okayama | 2005030 |
| | Local Independent Administrative Corporation Hiroshima City Hospital Organization Hiroshima City Hiroshima Citizens Hospital Institutional Review Board 7-33 Motomachi, Naka-ku, Hiroshima-shi, Hiroshima | 2005031 |

| Country | Name/Address of IRB/IEC | Site number |
|---|---|---|
| | Review Board of Human Rights and Ethics for Clinical Studies Institutional Review Board 13-2 Ichibancho, Chiyoda-ku, Tokyo | 2005032 |
| | Aijinkai Takatsuki General Hospital IRB 1-3-13 Kosobe-cho, Takatsuki, Osaka | 2005033 |
| | Review Board of Human Rights and Ethics for Clinical Studies Institutional Review Board 13-2, Ichibancho, Chiyoda-ku, Tokyo, Japan | 2005034 |
| | Japanese Red Cross Shizuoka Hospital Institutional Review Board 8-2 Otemachi, Aoi-ku, Shizuoka-shi, Shizuoka | 2005035 |
| | JA Shizuoka Kosei Hospital Institutional Review Board 23 Kitabancho, Aoi-ku, Shizuoka-shi, Shizuoka | 2005036 |
| | Hiroshima Red Cross Hospital & Atomicbomb Survivors Hospital Institutional Review Board 1-9-6 Sendamachi, Naka-ku, Hiroshima-shi | 2005037 |
| | NHO Shikoku Medical Center for Children and Adults Institutional Review Board 2-1-1, Senyucho, Zentsuji-shi, Kagawa, Japan | 2005038 |
| | Daido Hospital Institutional Review Board 9 Hakusuicho, Minami-ku, Nagoya, Aichi | 2005039 |
| | Nagoya Ekisaikai Hospital IRB, 4-66 Shonen-Cho, Nakagawa-ku, Nagoya-Shi, Aich | 2005049 |
| Republic of Korea | Samsung Medical Center Institutional Review Board | 2004768 |
| | Yonsei University Health system, Severance Hospital, Institutional review Board, Yonsei-ro 50-1, Seodaemun-gu, Seoul, 03722 | 2004769 |
| | IRB of Korea University Ansan Hospital, 123 Jeokgeum-ro (Gojan-dong) Danwon-gu, Ansan-si, Gyeonggi-do, 15355 | 2004797 |
| | Inha University Hospital Institutional Review Board, 27 Inhang-ro, Jung-gu, Incheon | 2004798 |
| | Yonsei University Gangnam Severance Hospital, IRB, 2nd Floor, 235 Dogok-ro, Gangnam-gu, Seoul 06230 | 2004800 |
| Latvia | Ethics Committee for Clinical Trials of Medicinal Products,Aizkraukles street 21 - 113, Riga,LV1006 | 2004380, 2004199, 2004331, 2004202, 2004198, 2004302 |
| Lithuania | Lithuanian Bioethics Committee,Algirdo g. 31, Vilnius,LT-03219 | 2004395, 2004204, 2004277, 2004710 |
| Mexico | Federico Gomez Children's hospital of Mexico, National Institute of Health research office | 2004404 |
| New Zealand | Northern B Health and Disability Ethics Committee,20 Aitken Street, Ministry of Health, Ethics Department, Reception - Ground Floor, Thorndon, Wellington,6011 | 2004335, 2004405, 2004048, 2004105 |
| Panama | Dr Jose Renan Esquivel Children's hospital, Panama Ave, Balboa, Calle 34 Research Bioethics Committee | 2004867, 2004868, 2004869, 2004870, 2004871, 2004872 |
| Poland | Komisja Bioetyczna przy Okregowej Izbie Lekarskiej w Rzeszowie,ul. Jana Dekerta 2, Rzeszów,35- 030 | 2004674, 2004371, 2004049, 2004334, 2004206, 2004381, 2004205, 2004208, 2004350, 2004305 |
| Russian Federation | St. Petersburg State Budgetary Institution of Healthcare 'Children's Municipal Polyclinic No. 35' 168 Bldg. 2 Leninskiy Ave., St. Petersburg 196191 | 2004336, 2004106 |
| | Medical Technologies LLC; 6 Nevzorovoy Str., St. Petersburg 192148; | 2004212 |

| Country | Name/Address of IRB/IEC | Site number |
|---|---|---|
| | State Budgetary Healthcare Institution of the Perm Territory ‘Municipal Children’s Clinical Polyclinic No. 5’ | 2004214 |
| South Africa | Pharma Ethics Independant Research Erthics committee,123 Amcor Road,Lyttelton Manor Pretoria,0157 | 2004034, 2004108, 2004110, 2004712 |
| | Wits Health Consortium,31 Princess of Wales Terrace,Parktown Johannesburg,2193 | 2004217, 2004109, 2004216 |
| | 1 Military Hospital Human Research Ethics Committee, Department of Neurology Private bag X 1026 Thaba Tswane 0143 | 2004111 |
| | Stellenbosch University Human Research Ethics Committee, Stellenbosch University Private Bag X1, Matieland, 7602, Stellenbosch, South Africa | 2004039 |
| | University of Cape Town Human Research Ethics Committee, DEPARTMENT OF PAEDIATRICS AND CHILD HEALTH RED CROSS WAR MEMORIAL CHILDREN’S HOSPITAL KLIPFONTEIN ROAD RONDEBOSCH 7700 | 2004036 |
| Spain | Hospital Universitario Clinico San Carlos,Puerta G - Planta 4ª Norte,C/ Profesor Martin Lagos, s/n Madrid,28040 | 2004033, 2004112, 2004113, 2004114, 2004115, 2004218, 2004219, 2004311, 2004333, 2004344, 2004363, 2004369, 2004382, 2004385, 2004406, 2004675, 2004407, 2005603 |
| Sweden | Etikprövningsmyndighete n,Box 2110,SE-750 02 Uppsala,SE-750 02 | 2004402, 2004116 |
| Turkey | Ege University Ethics Committee,Ege Universitesi Tip Fakultesi,Klinik Arastirmalar Etik Kurulu Izmir,35100 | 2004222, 2004221, 2004223, 2004224 |
| Ukraine | Dept of health of Chernivtsi city council, Communal Medical Institution City Clinical Childrens' Hospital, 4 Bukovynska St, Chernivtsi, 58001 | 2004117 |
| | Ministy of Health of Ukrain, Communal Non-Commercial enterprise Saint Zinaida Children's Clinical Hospital of Sumy City Council, 28 Troiiska st, Sumy, 40022 | 2004227 |
| | Vinnytsia regional Children's Clinical Hospital, 108 Khmelnytske shose st, Vinnytsia, 21000. Medical Ethics Commission | 2004229 |
| | State Institution Academician O.M Lukyanova Institute of Pediatrics, obstetrics and gynecology of national academy of medical sciences of Ukraine, 8 P.Mayborody str Kyiv, 04050 | 2004294 |
| | Communal Non-Commercial enterprise of Kharkiv Regional Council regional Children's clinical hospital, 5 Ozeryanska st Kharkiv, 61093 | 2004322 |
| | Odesa Regional State administration, department of health, communal enterprise, Odesa regional Children's clinical hospital, 3 Ac Vorobiov st, Odes-31, 65031 | 2004338 |
| | Ethics Commission at Communal Institution Dnipro City Children's Clinical Hospital No 5 of Dnipro City Council, 5 ivana Akinfiieva st, Dnipro 49027 Ukraine | 2004633 |
| United Kingdom | NRES Committee South Central - Berkshire, South West REC Centre, Level 3, Block B Bristol,BS1 2NT | 2004355, 2004384, 2004682, 2004689, 2004383 |

| Country | Name/Address of IRB/IEC | Site number |
|---|---|---|
| United States | WCG IRB 212 Carnegie Center, Suite 301 Princeton, NJ 08540, USA | 2004023, 2004025, 2004027, 2004028, 2004030, 2004031, 2004032, 2004118, 2004236, 2004237, 2004239, 2004240, 2004243, 2004253, 2004255, 2004256, 2004258, 2004259, 2004260, 2004261, 2004263, 2004264, 2004267, 2004268, 2004278, 2004279, 2004280, 2004291, 2004292, 2004293, 2004314, 2004315, 2004316, 2004319, 2004323, 2004340, 2004345, 2004376, 2004386, 2004389, 2004394, 2004409, 2004613, 2004614, 2004615, 2004616, 2004618, 2004624, 2004634, 2004650, 2004652, 2004656, 2004657, 2004664, 2004677, 2004679, 2004680, 2004690, 2004697, 2004699, 2004700, 2004702, 2004746, 2004873, 2005604, 2005605, 2005606 |
| | The University of Oklahoma, Institutional Review Board for the Protection of Human Subjects, 1105N. Stone wall Avenue, Oklahoma City, OK73117(FWA 007961) | 2004026 |
| | Nemours Office of Human Subjects Protection Nemours/Alfred I. duPont Hospital for Children 1600 Rockland Road Wilmington, DE 19803 | 2004029 |
| | Institutional Review Board Ann & Robert H. Lurie Children's Hospital of Chicago, 25 East Chicago Avenue, Chicago, Illinois | 2004238 |
| | Cincinnati Children’s Hospital Institutional Review Board, 3333 Burnet Avenue \| MLC 7040 \| Cincinnati, OH 45229 | 2004241 |
| | MetroHealth Institutional Review board, 2500 MetroHealth Drive, Cleveland Ohio 44109 | 2004281 |
| | Creighton University office of the provost Research Compliance, 2500 California Plaza Omaha, NE 68178-0001 | 2004310 |
| | Medical University of South Carolina, 179 Ashley Ave, Charleston, SC 29425 | 2004341 |
| | University of Nebraska Medical Center, 42nd and Emile Streets Omaha, NE 68198 402-559-4000 | 2004391 |
| | Nationwide Children's IRB, Nationwide Children’s Hospital 700 Childrens Drive Columbus, OH 43205 | 2004658 |
| | The University Of Tennessee, Health Science Centre Institutional Review Board 910 Madison Avenue, Suite 600 Memphis, TN 38163 | 2004662 |
| | Marshfield Clinic Research Instiitute Institutional Review Board 1000N, Oak Ave Marshfield, WI 54449-5790 | 2004678 |
| | EMORY UNIVERSITY Institutional Review Board 201 Dowman Dr, Atlanta, GA 30322, United States | 2004708 |
| | Navajo Nation Human Research Review Board Navajo Division of Health, P. O. Box 1390, Window Rock, AZ 86515 | 2004747, 2004748, 2004749 |

**Figure S1:** Participant disposition (CONSORT diagram): healthy preterm and term infants in the Phase 2b and MELODY trials.

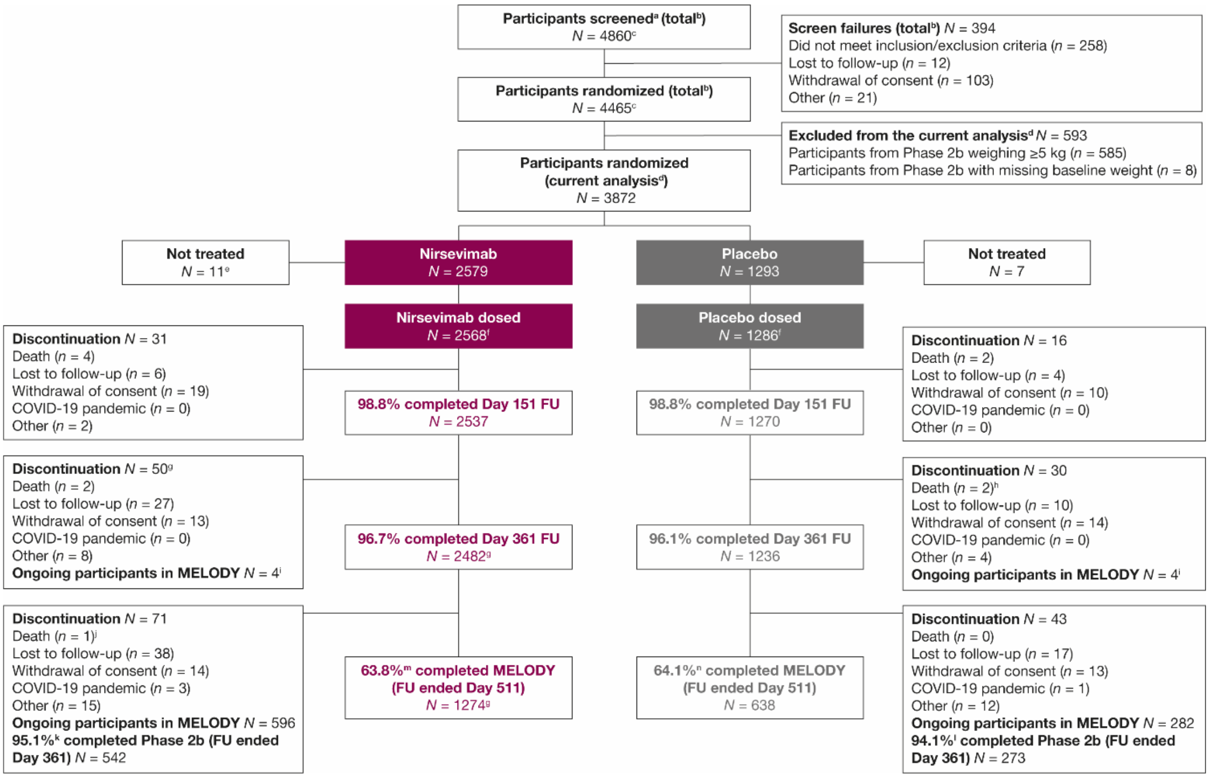


Phase 2b final database lock: February 14, 2019. MELODY data cut-off: November 9, 2022.

[a]Informed consent signed.

[b]Includes all participants from the Phase 2b study and MELODY full enrollment cohort.

[c]One participant from MELODY was randomized twice in the study, was assigned two different subject IDs, and received two doses of nirsevimab 100 mg. One of the two subject IDs was excluded from participants randomized to avoid double counting the same individual in the analysis.

[d]Includes infants from Phase 2b weighing <5 kg and the full MELODY enrollment cohort.

[e]Includes one death of a randomized participant who never received nirsevimab.

[f]Two participants randomized to placebo incorrectly received nirsevimab; both participants were included in the as-treated population under the nirsevimab group. As-treated population: placebo (*N* = 1284), nirsevimab (*N* = 2570).

[g]One participant did not complete Day 361 and mistakenly had their end of a study status recorded as completed; this participant is not included among the discontinuations.

[h]Includes one participant from Phase 2b who did not complete the Day 361 visit, was considered an early discontinuation, and died on Day 367 due to bronchopneumonia; this death was considered unrelated to nirsevimab and is not included within the safety analysis.

[i]Four participants in each treatment group completed the Day 361 visit within the allowed 7-Day window but had not completed chronological Day 361 at the time of this safety analysis.

[j]One participant in MELODY died on Day 440 due to an automobile accident; this death is not included within the safety analysis.

[k]Based on 570 participants from Phase 2b weighing <5 kg who were planned to receive nirsevimab and were included in this analysis.

[l]Based on 290 participants from Phase 2b weighing <5 kg who were planned to receive placebo and were included in this analysis.

[m]Based on 1998 participants who received nirsevimab in MELODY.

[n]Based on 996 participants who received placebo in MELODY.

Abbreviations: CONSORT, Consolidated Standards of Reporting Trials; COVID-19, coronavirus disease 2019; FU, follow-up.

**Figure S2:** Participant disposition (CONSORT diagram): infants with CHD/CLD and preterm infants born ≤35 weeks 0 days GA without CHD/CLD in the MEDLEY trial.

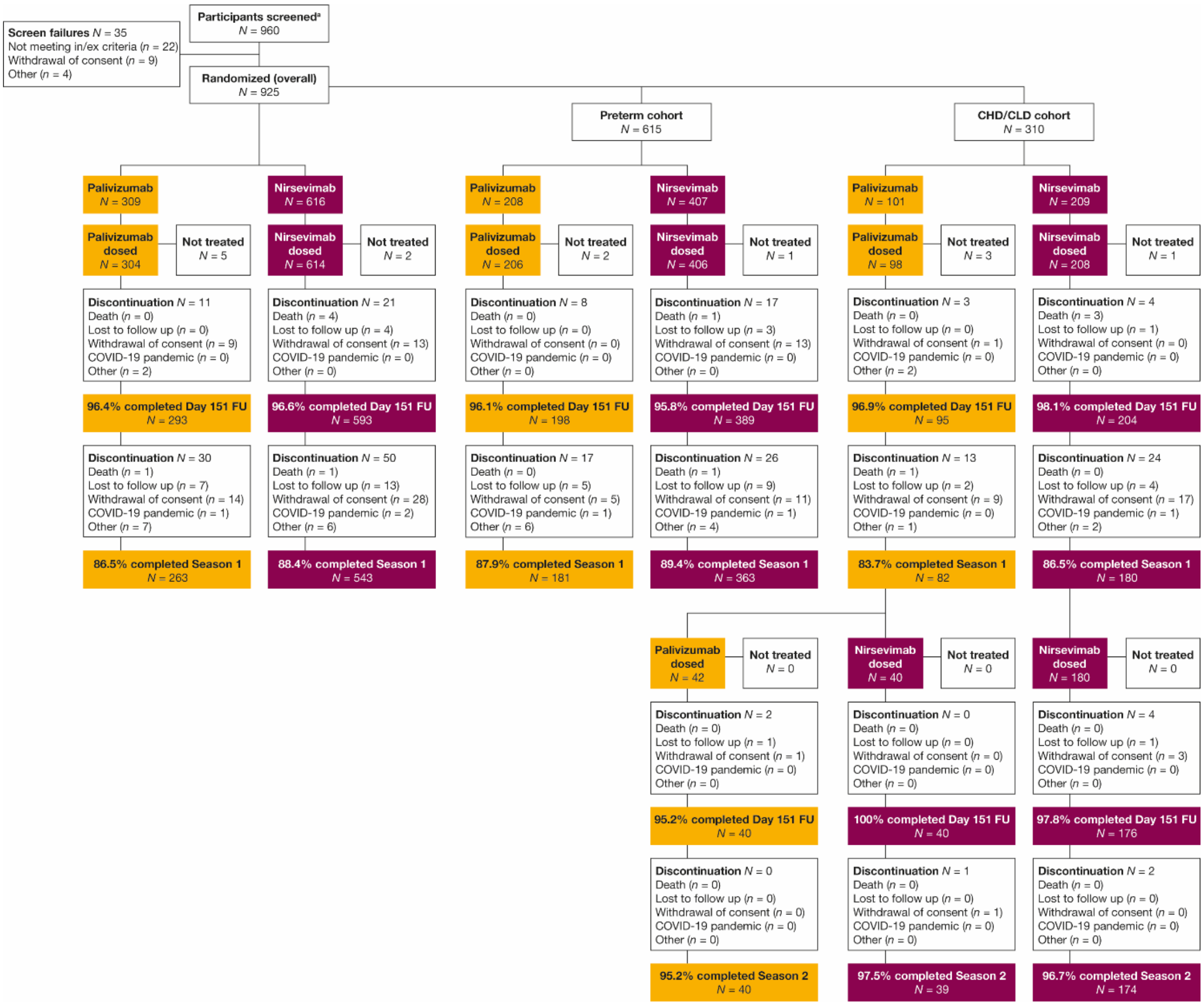


Final database lock: February 22, 2023.
[a]Informed consent signed.
Abbreviations: CONSORT, Consolidated Standards of Reporting Trials; COVID-19, coronavirus disease 2019; FU, follow-up.

**Figure S3:** Summary of AEs through 360 days post-dose in healthy term and preterm infants born ≥29 wGA who were (A) neonates or (B) weighed <2.5 kg at dosing[a] and infants at higher risk of severe disease eligible for palivizumab who were (C) born <29 wGA,[b] (D) with CHD,[c] or (E) with CLD[c].

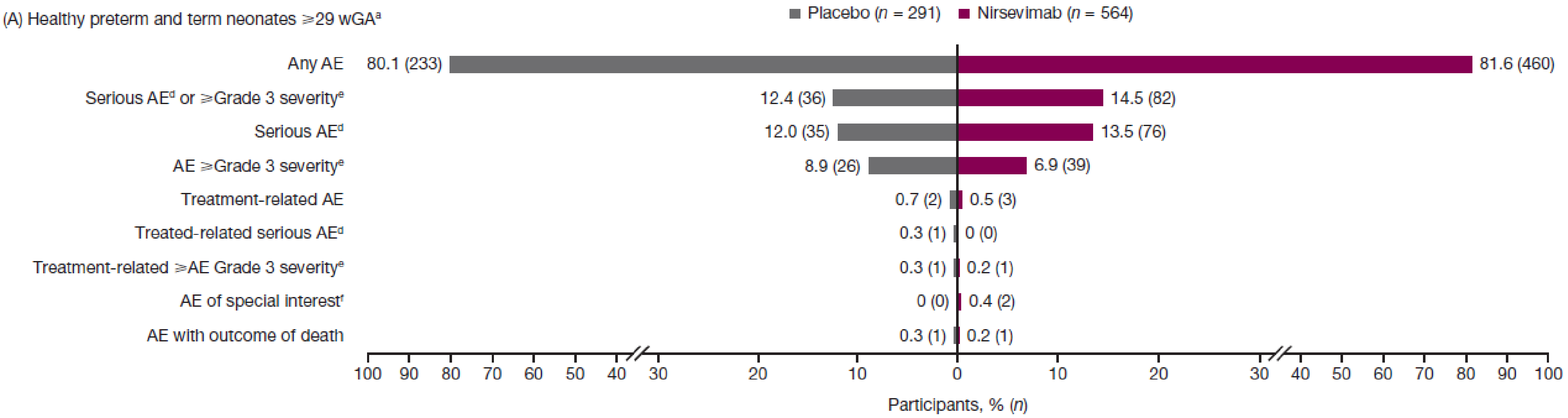


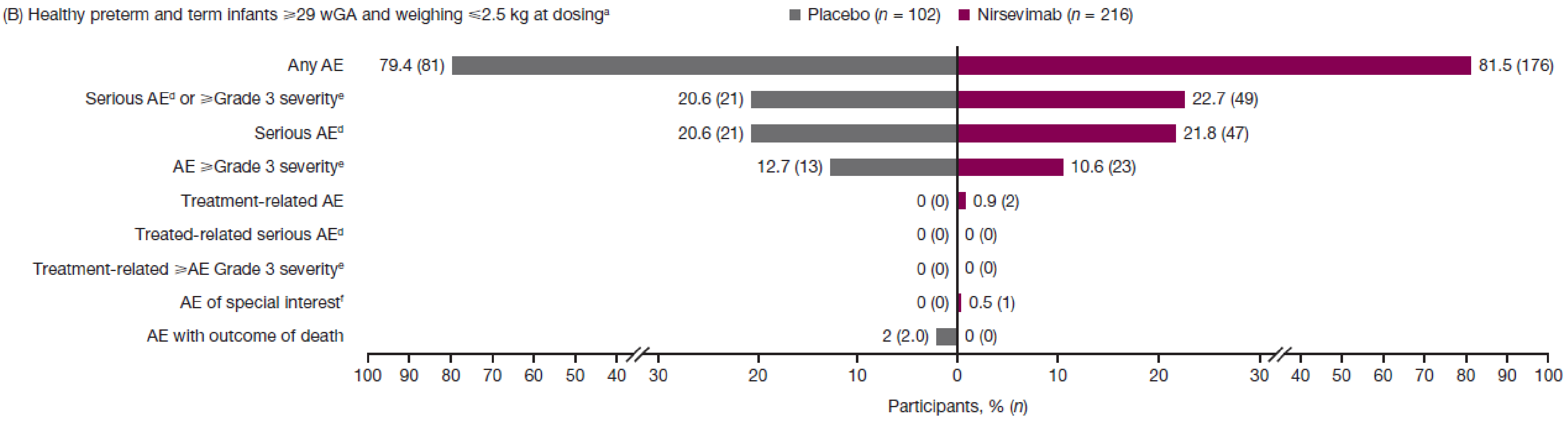

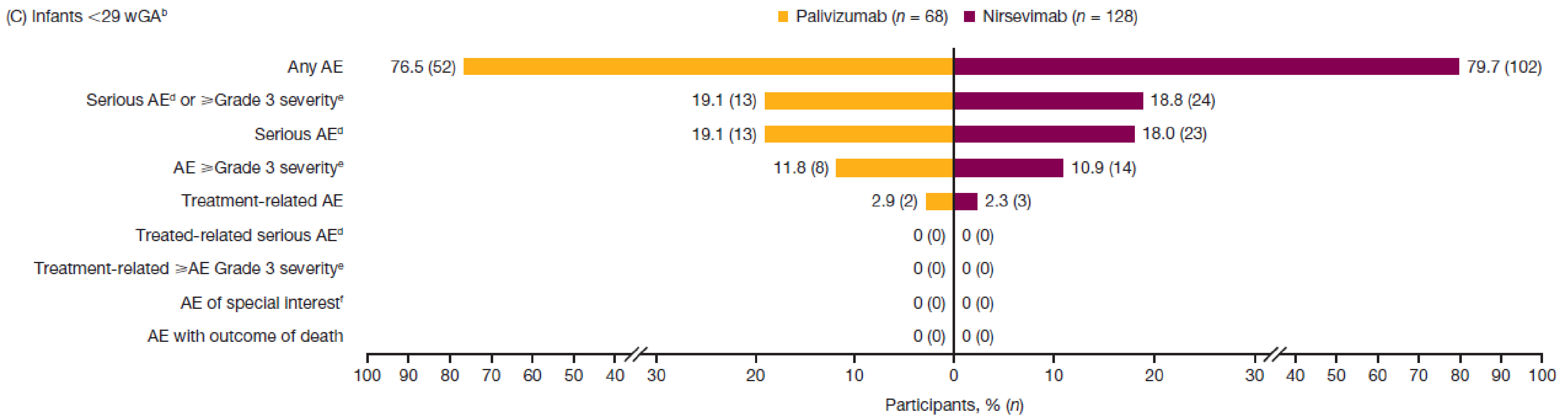

(C) Infants <29 wGA[b]
Palivizumab (n = 68) Nirsevimab (n = 128)
Any AE
Serious AE[d] or ≥Grade 3 severity[e]
Serious AE[d]
AE ≥Grade 3 severity[e]
Treatment-related AE
Treated-related serious AE[d]
Treatment-related ≥AE Grade 3 severity[e]
AE of special interest[f]
AE with outcome of death
76.5 (52)
79.7 (102)
19.1 (13)
18.8 (24)
19.1 (13)
18.0 (23)
11.8 (8)
10.9 (14)
2.9 (2)
2.3 (3)
0 (0)
100 90 80 70 60 50 40 30 20 10 0 10 20 30 40 50 60 70 80 90 100
Participants, % (n)


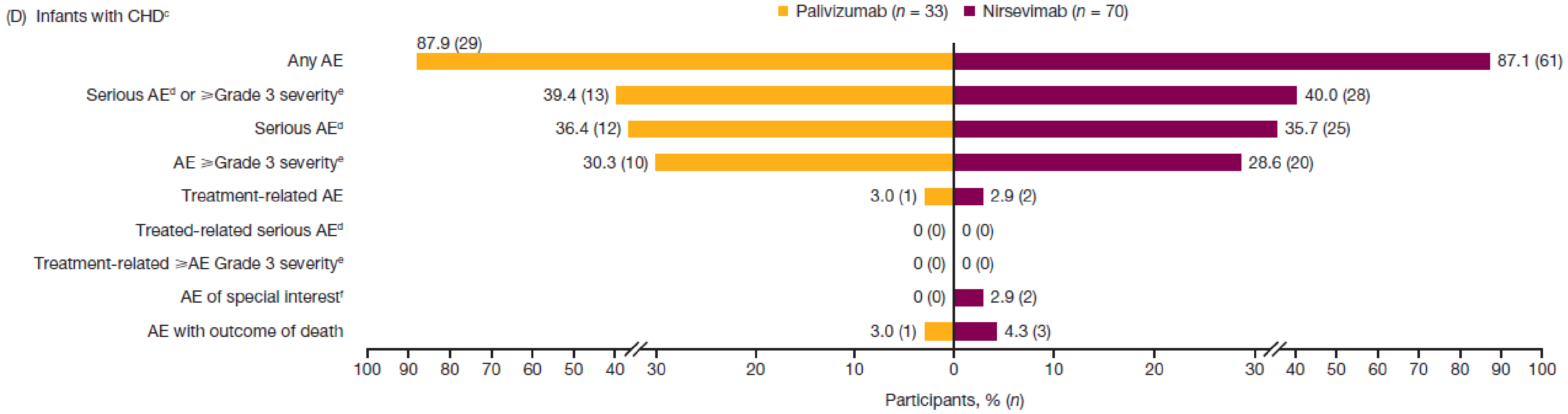

(D) Infants with CHD[c]
Palivizumab (n = 33) Nirsevimab (n = 70)
Any AE
Serious AE[d] or ≥Grade 3 severity[e]
Serious AE[d]
AE ≥Grade 3 severity[e]
Treatment-related AE
Treated-related serious AE[d]
Treatment-related ≥AE Grade 3 severity[e]
AE of special interest[f]
AE with outcome of death
87.9 (29)
87.1 (61)
39.4 (13)
40.0 (28)
36.4 (12)
35.7 (25)
30.3 (10)
28.6 (20)
3.0 (1)
2.9 (2)
0 (0)
4.3 (3)
100 90 80 70 60 50 40 30 20 10 0 10 20 30 40 50 60 70 80 90 100
Participants, % (n)

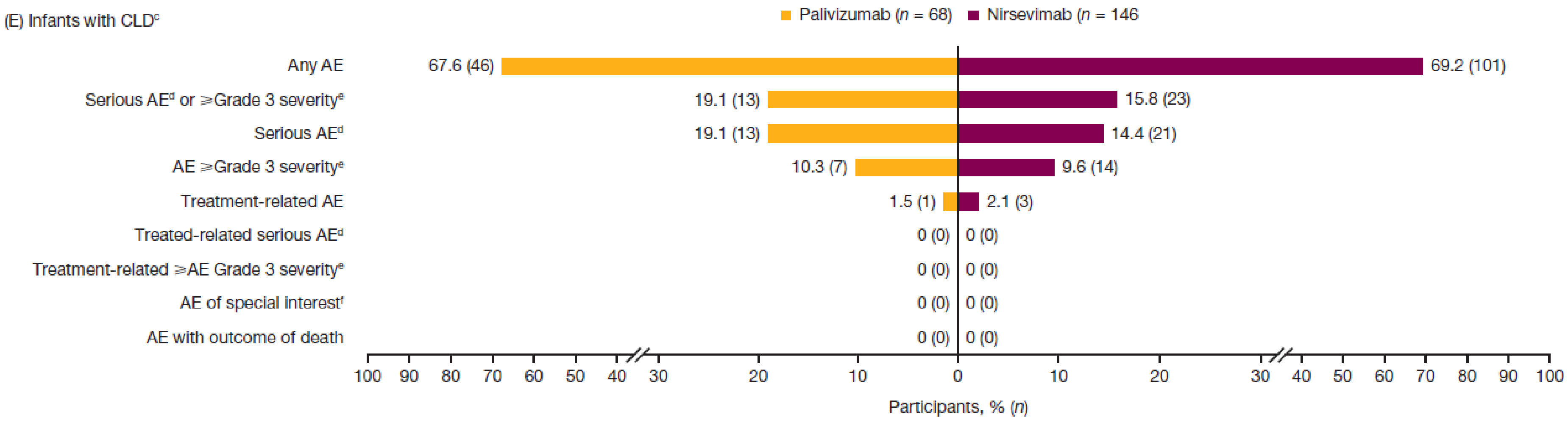


Participants with multiple events in the same category were counted once in that category; participants with events in >1 category were counted once in each category.
[a]Includes infants from Phase 2b weighing <5kg and the full MELODY enrollment cohort.
[b]Includes participants from the MEDLEY Season 1 preterm cohort and CHD/CLD cohort born <29 wGA. [c]Infants with both CHD and CLD were included in both the CHD and the CLD subpopulations.
[d]Defined as death, life-threatening, requiring inpatient hospitalization, prolongation of existing hospitalization, persistent or significant disability/incapacity, important medical event, congenital anomaly/birth defect.
[e]Grade 1: mild, Grade 2: moderate; Grade 3: severe; Grade 4: life-threatening; Grade 5: fatal.
[f]Included immediate type I hypersensitivity reactions (eg, anaphylaxis), immune complex disease, and thrombocytopenia.
Abbreviations: AE, adverse event; CHD, congenital heart disease; CLD, chronic lung disease; wGA, weeks gestational age.

**Figure S4:** Summary of AEs within 30 days of dosing in (A) preterm infants born ≤35 weeks 0 days GA without CHD/CLD or (B) infants with CHD/CLD entering their first RSV season and (C) children with CHD/CLD entering their second RSV season[a].

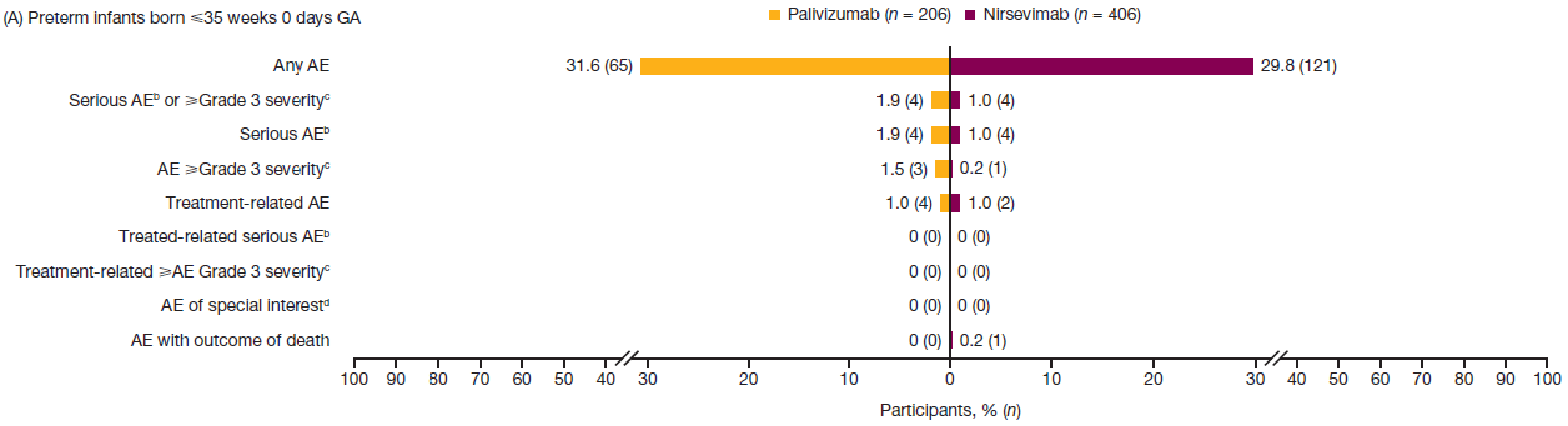


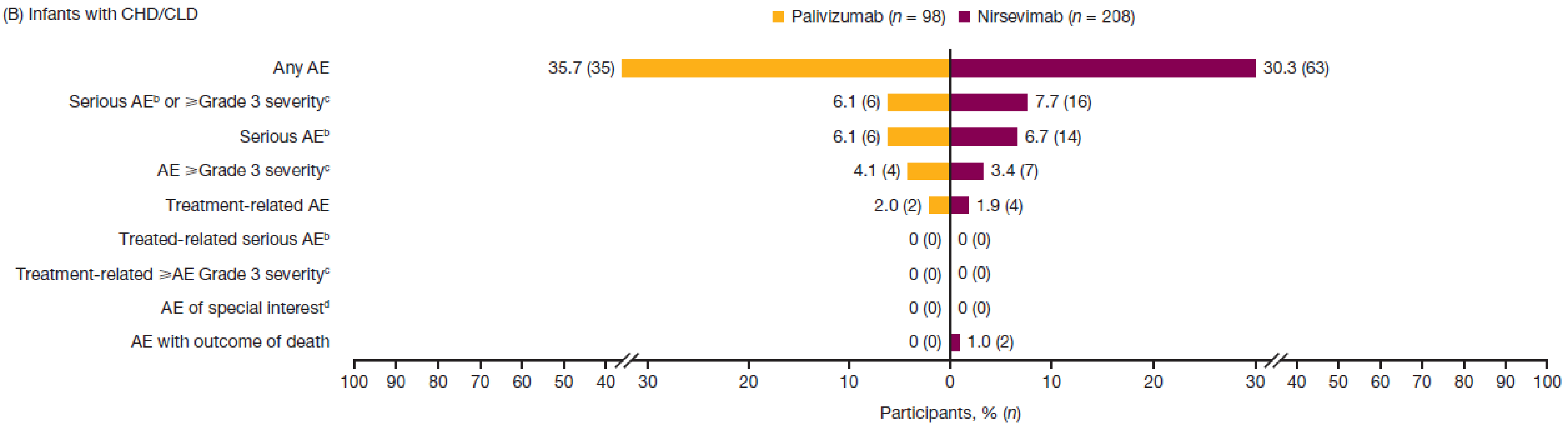

(C) Children with CHD/CLD entering their second RSV season[a]

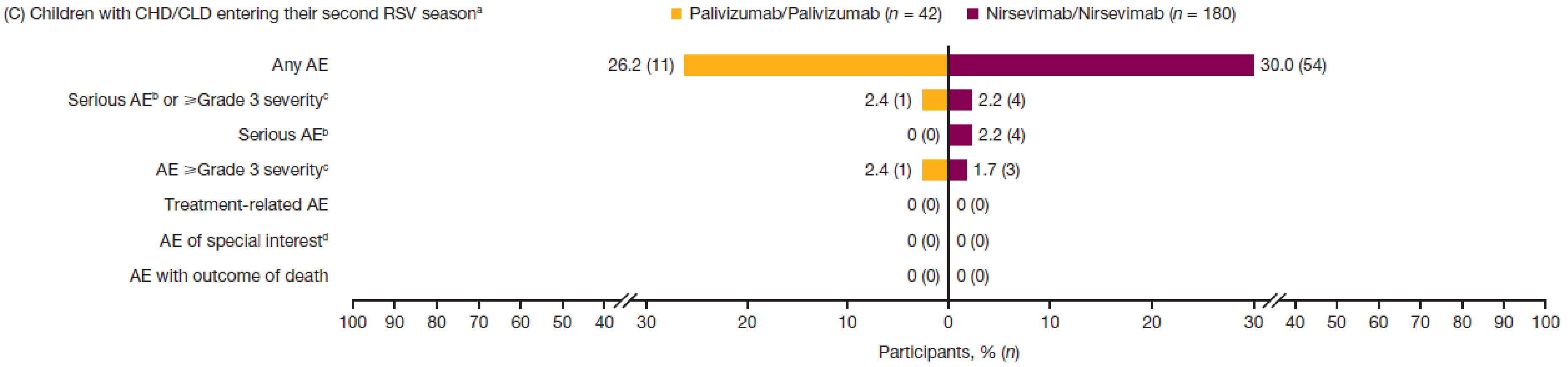


Participants with multiple events in the same category were counted once in that category; participants with events in >1 category were counted once in each category.
[a]Before the second season, children with CHD/CLD randomized to nirsevimab in the first season received a single IM dose of 200 mg nirsevimab followed by 4x once-monthly IM doses of placebo (nirsevimab/nirsevimab) and those randomized to palivizumab in the first season were re-randomized 1:1 to either a single IM dose of 200 mg nirsevimab followed by 4x once-monthly IM doses of placebo (palivizumab/nirsevimab) or 5x once-monthly IM doses of palivizumab (15 mg/kg per dose; palivizumab/palivizumab).
[b]Defined as death, life-threatening, requiring inpatient hospitalization, prolongation of existing hospitalization, persistent or significant disability/incapacity, important medical event, congenital anomaly/birth defect.
[c]Grade 1: mild; Grade 2: moderate; Grade 3: severe; Grade 4: life-threatening; Grade 5: fatal.
[d]Included immediate type I hypersensitivity reactions (eg, anaphylaxis), immune complex disease, and thrombocytopenia.
Abbreviations: AE, adverse event; CHD, congenital heart disease; CLD, chronic lung disease; GA, gestational age; IM, intramuscular; RSV, respiratory syncytial virus.

**Figure S5:** Summary of AEs in children with CHD/CLD entering their second RSV season through (A) 360 days and (B) 30 days post first dose in the second season[a].

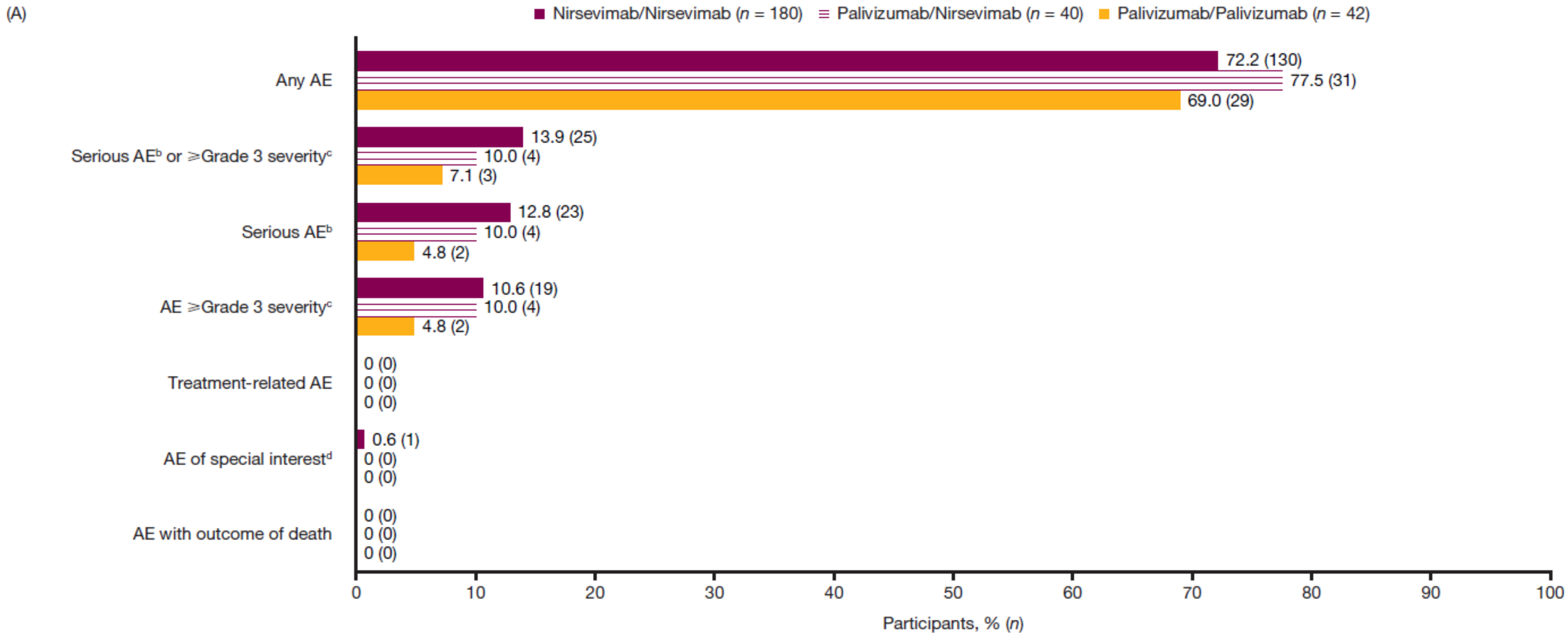

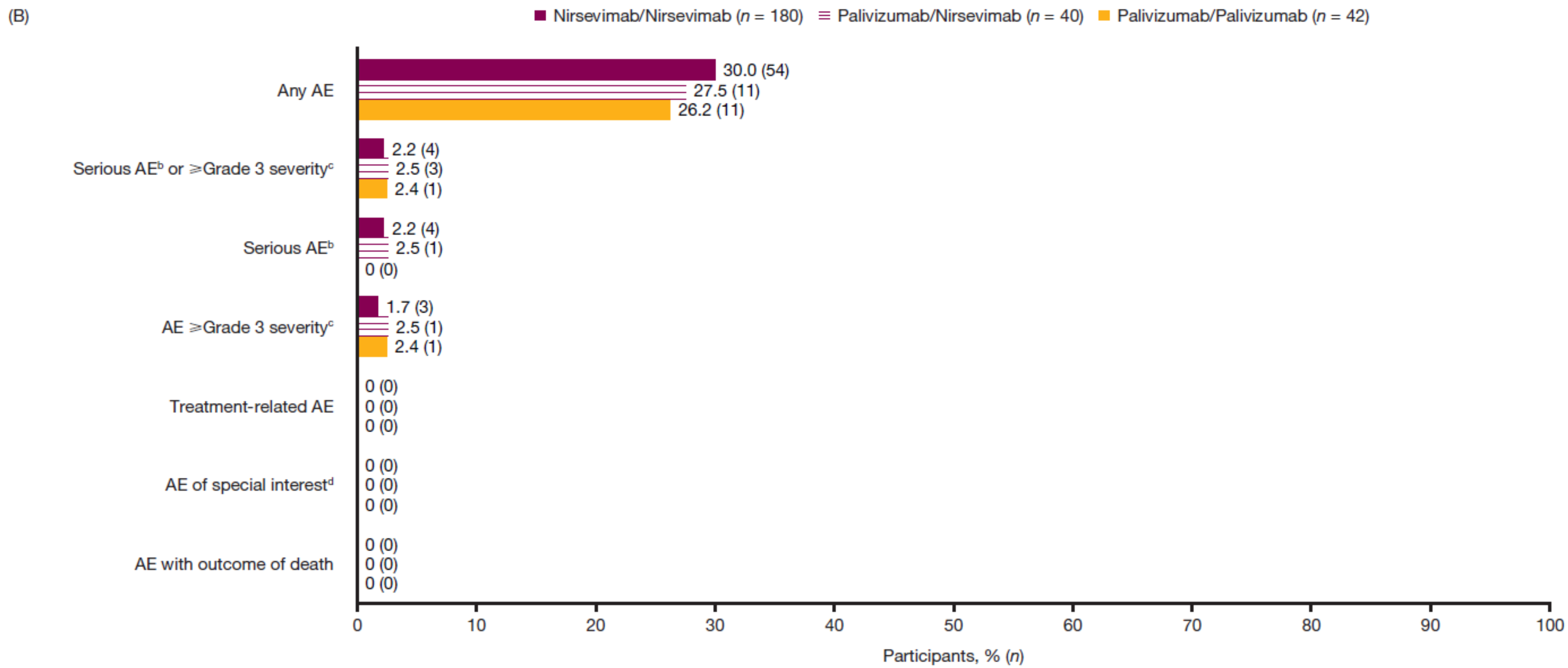


[a]Before the second season, children with CHD/CLD randomized to nirsevimab in the first season received a single IM dose of 200 mg nirsevimab followed by 4x once-monthly IM doses of placebo (nirsevimab/nirsevimab) and those randomized to palivizumab in the first season were re-randomized 1:1 to either a single IM dose of 200 mg nirsevimab followed by 4x once-monthly IM doses of placebo (palivizumab/nirsevimab) or 5x once-monthly IM doses of palivizumab (15 mg/kg per dose; palivizumab/palivizumab).
[b]Defined as death, life-threatening, requiring inpatient hospitalization, prolongation of existing hospitalization, persistent or significant disability/incapacity, important medical event, congenital anomaly/birth defect.
[c]Grade 1: mild; Grade 2: moderate; Grade 3: severe; Grade 4: life-threatening; Grade 5: fatal.
[d]Included immediate type I hypersensitivity reactions (eg, anaphylaxis), immune complex disease, and thrombocytopenia.
Abbreviations: AE, adverse event; CHD, congenital heart disease; CLD, congenital lung disease; IM, intramuscular; RSV, respiratory syncytial virus.

**Figure S6:** Nirsevimab serum $AUC_{0-365}$ (A and B) and $C_{max}$ (C and D) in participants with ≥Grade 3 AEs[a] or serious AEs[b] (A and C) and AESI[c] (B and D).

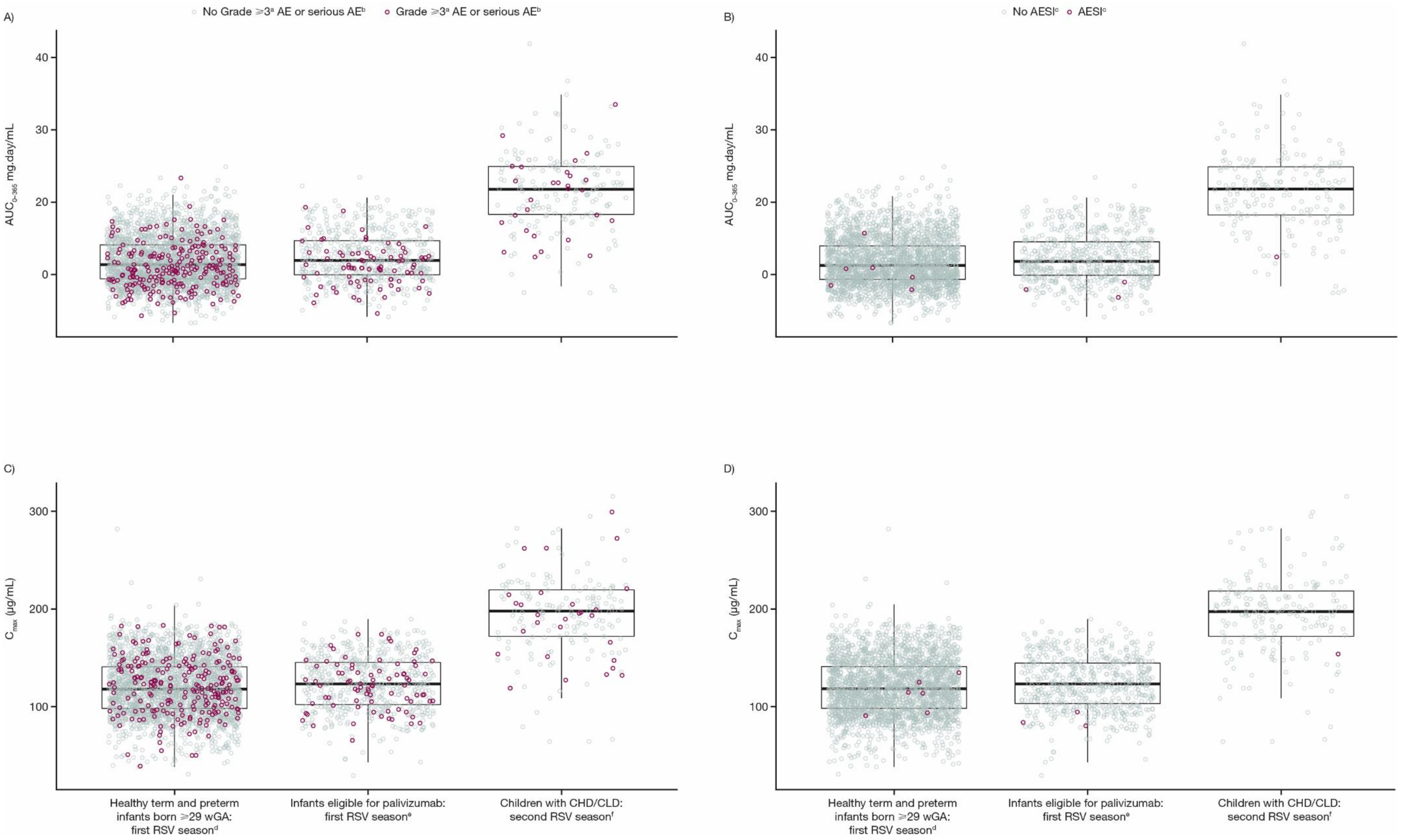

[a]Grade 1: mild; Grade 2: moderate; Grade 3: severe; Grade 4: life-threatening; Grade 5: fatal.
[b]Defined as death, life-threatening, requiring inpatient hospitalization, prolongation of existing hospitalization, persistent or significant disability/incapacity, important medical event, congenital anomaly/birth defect.
[c]Included immediate type I hypersensitivity reactions (eg, anaphylaxis), immune complex disease, and thrombocytopenia.
[d]Includes infants from Phase 2b weighing <5kg and the full MELODY enrollment cohort.
[e]Includes infants with CHD and/or CLD or infants born ≤35 weeks 0 days GA without CLD or CHD.
[f]Includes participants from the nirsevimab/nirsevimab and palivizumab/nirsevimab groups. Before the second season, children with CHD/CLD randomized to nirsevimab in the first season received a single IM dose of 200 mg nirsevimab followed by 4x once-monthly IM doses of placebo (nirsevimab/nirsevimab) and those randomized to palivizumab in the first season were re-randomized 1:1 to either a single IM dose of 200 mg nirsevimab followed by 4x once-monthly IM doses of placebo (palivizumab/nirsevimab) or 5x once-monthly IM doses of palivizumab (15 mg/kg per dose; palivizumab/palivizumab).
Abbreviations: AE, adverse event; AESI, adverse event of special interest; $AUC_{0–365}$, area under the concentration-time curve from Day 0 to Day 365; CHD, congenital heart disease; CLD, chronic lung disease; $C_{max}$, maximum serum concentration; IM, intramuscular; RSV, respiratory syncytial virus; wGA, weeks gestational age.

**Table S1:** Treatment Exposure and Follow-Up.

| Safety Analysis Population | Extent of Exposure | Participants with Safety Follow-Up Through 360 Days Post-Dose, % |
|---|---|---|
| Healthy term and preterm infants born ≥29 wGA[a]<br>Phase 2b/MELODY | • 3854 participants received nirsevimab (*n* = 2570) or placebo (*n* = 1284)<br>– Received 50 mg dose: 53.6% of nirsevimab recipients<br>– Received 100 mg dose: 46.4% of nirsevimab recipients | 96.5% |
| Infants with CHD/CLD or preterm infants born ≤35 weeks 0 days GA without CHD/CLD<br>MEDLEY Season 1 | • Nirsevimab (*n* = 614): All received at least 1 active dose[b]<br>– Received 50 mg dose: 56.2% of nirsevimab recipients[c]<br>– Received 100 mg dose: 43.6% of nirsevimab recipients[c,d]<br>• Palivizumab (*n* = 304): 90.5% received at least 5 active doses[b] | 87.8 % |
| Children with CHD/CLD entering their second RSV season[e]<br>MEDLEY Season 2 | • Nirsevimab/nirsevimab (*n* = 180): All received at least 1 active dose[b]<br>• Palivizumab/nirsevimab (*n* = 40): All received at least 1 active dose[b]<br>• Palivizumab/palivizumab (*n* = 42): 90.5% received at least 5 active doses[b] | 96.6% |

[a]Includes infants from Phase 2b weighing <5 kg and the full MELODY enrollment cohort.
[b]Participants with CHD could receive an active dose following cardiac surgery with cardiopulmonary bypass.
[c]Refers to the first dose of Season 1.
[d]A single participant weighing 6.1 kg received less than the intended volume due to syringe malfunction and received an estimated dose of 67 mg instead of 100 mg (recorded as a protocol deviation).
[e]Before the second season, children with CHD/CLD randomized to nirsevimab in the first season received a single IM dose of 200 mg nirsevimab followed by 4x once-monthly IM doses of placebo (nirsevimab/nirsevimab) and those randomized to palivizumab in the first season were re-randomized 1:1 to either a single IM dose of 200 mg nirsevimab followed by 4x once-monthly IM doses of placebo (palivizumab/nirsevimab) or 5x once-monthly IM doses of palivizumab (15 mg/kg per dose; palivizumab/palivizumab).
Abbreviations: CHD, congenital heart disease; CLD, chronic lung disease of prematurity; GA, gestational age; IM, intramuscular; RSV, respiratory syncytial virus; wGA, weeks gestational age.

**Table S2:** AESI Among Nirsevimab Recipients Across Pivotal Trials Through 360 Days Post-Dose.

| Trial | Relationship to Treatment | Toxicity Grade[a]/ Seriousness[b] | Study Day of AE onset | MedDRA Preferred Term | Description | Days to Resolution[c] |
|---|---|---|---|---|---|---|
| **AESI of hypersensitivity** | | | | | | |
| Phase 2b | Related | Grade 1/nonserious | Day 3 | Rash | Maculopapular rash with generalized and symmetrical distribution on neck and trunk | 13 days |
| Phase 2b | Related | Grade 1/nonserious | Day 120 | Petechiae | Based on parental description of skin lesions with a generalized distribution on face, trunk, and arms; no laboratory or healthcare provider assessment | 2 days |
| MELODY | Related | Grade 1/nonserious | Day 1 | Rash papular | Erythematous maculopapular rash with generalized distribution on face, trunk, arms, hands and legs | 6 days |
| MELODY | Related | Grade 1/nonserious | Day 1 | Rash maculopapular[d] | Erythematous maculopapular rash with symmetrical distribution on legs | 1 Day |
| MELODY | Related | Grade 1/nonserious | Day 1 | Rash maculopapular | Erythematous maculopapular rash with a generalized distribution on head, face, neck, trunk, arms, and legs; treated with oral antihistamine | 3 days |
| MELODY | Related | Grade 3/nonserious | Day 7 | Rash | Macular rash with a generalized distribution on head, face, neck, trunk, arms, hands, legs, feet, buttocks/groin; exposure to new probiotic | 20 days |
| MEDLEY Season 1 (preterm cohort) | Related | Grade 1/nonserious | Day 93[e] after a dose of placebo | Rash maculopapular | Maculopapular rash with a generalized distribution on face and trunk following a dose of placebo for nirsevimab; treatment withdrawn | 1 Day |
| **AESI of Thrombocytopenia** | | | | | | |
| MEDLEY Season 1 CHD (hypoplastic | Not related | Grade 2/nonserious | Day 52 | Heparin-induced thrombocytopenia | Thrombocytopenia following heparin administration for cardiac catheterization for stent angioplasty of aortic coarctation in an infant with CHD who also received a dose of palivizumab outside the study (Day 22) prior to | 5 days |

| | | | | | | |
|---|---|---|---|---|---|---|
| left heart syndrome[f]) | | | | | the event. Platelet nadir 23,000/µL 4 days after heparin administration; recovery to 202,000/µL following platelet transfusion | |
| MEDLEY Season 1 CHD (VSD,[f] ASD, Down syndrome) | Not related | Grade 1/nonserious | Day 40 | Thrombocytopenia | Reported as thrombocytopenia due to nosocomial sepsis (on same Day as event of sepsis) during hospitalization for cardiac failure complicated by nosocomial pneumonia (non-RSV), sepsis, and ultimately death (Day 66) from cardiogenic shock following urgent surgery for repair of atrioventricular septal defect. No treatment for thrombocytopenia | 12 days |
| MEDLEY Season 2 CHD (hypoplastic left heart syndrome[f]) | Not related | Grade 2/nonserious | Day 253 | Pancytopenia | Thrombocytopenia and leukopenia in the context of aseptic meningitis due to roseola with confirmed Human Herpesvirus 6 infection (CSF sample). Platelet count at presentation (Day 253) was 59,000 / µL. Platelet transfusion was required (Day 254) after which platelet count recovered to 105,000 / µL at the time of discharge (Day 256). The WBC count was 1800 /µL (reference range 4000 – 12,000 /µL) at the time of the event and 3600 /µL at discharge | 78 days |

[a]Grade 1: mild, Grade 2: moderate; Grade 3: severe; Grade 4: life-threatening; Grade 5: fatal.
[b]Defined as death, life-threatening, requiring inpatient hospitalization, prolongation of existing hospitalization, persistent or significant disability/incapacity, important medical event, congenital anomaly/birth defect.
[c]Total number of days to resolution includes Day of onset.
[d]Participant who developed antidrug antibodies to nirsevimab which were first detected at the Day 361 visit (actual Study Day 393).
[e]Participant received active dose of nirsevimab Day 1 per protocol and mistakenly received a second active dose of nirsevimab at Day 31, recorded as a protocol deviation.
[f]Primary cardiac lesion as reported by the investigator.
Abbreviations: AE, adverse event; AESI, adverse event of special interest; ASD, atrial septal defect; CHD, congenital heart disease; CSF, cerebrospinal fluid; MedDRA, Medical Dictionary for Medical Activities; RSV, respiratory syncytial virus; VSD, ventricular septal defect; WBC, white blood cell.

**Table S3:** Most Common AEs (≥1% in Any Group) by Preferred Term within 14 Days Post-Dosing with or without Co-Administered Vaccine[a] in Healthy Term and Preterm Infants Born ≥29 wGA[b]

| | **Nirsevimab (*n* = 2570)** | | **Placebo (*n* = 1284)** | |
|---|---|---|---|---|
| **AEs, *n* (%)** | **With Vaccine (*n* = 695)** | **Without Vaccine (*n* = 1875)** | **With Vaccine (*n* = 374)** | **Without Vaccine (*n* = 910)** |
| Upper respiratory tract infection | 26 (3.7) | 64 (3.4) | 17 (4.5) | 19 (2.1) |
| Nasal congestion | 20 (2.9) | 24 (1.3) | 6 (1.6) | 11 (1.2) |
| Nasopharyngitis | 15 (2.2) | 38 (2.0) | 13 (3.5) | 22 (2.4) |
| Rhinitis | 14 (2.0) | 37 (2.0) | 13 (3.5) | 25 (2.7) |
| Pyrexia | 11 (1.6) | 15 (0.8) | 2 (0.5) | 7 (0.8) |
| Dermatitis diaper | 9 (1.3) | 23 (1.2) | 2 (0.5) | 9 (1.0) |
| Diarrhea | 5 (0.7) | 19 (1.0) | 6 (1.6) | 6 (0.7) |
| Seborrheic dermatitis | 3 (0.4) | 6 (0.3) | 4 (1.1) | 0 |
| Constipation | 2 (0.3) | 6 (0.3) | 1 (0.3) | 9 (1.0) |
| Irritability | 2 (0.3) | 5 (0.3) | 4 (1.1) | 3 (0.3) |

Participants with multiple events in the same preferred term were counted once in each of those preferred terms. Participants with events in more than one preferred term were counted once in each of those preferred terms.

[a]Co-administration within 14 days before or after nirsevimab or placebo with any of 7 pre-specified vaccine groups: polyvalent diphtheria-pertussis-tetanus containing vaccine, measles/mumps/rubella/varicella vaccine, rotavirus vaccine, pneumococcal vaccine, tuberculosis vaccine, hepatitis B vaccine, or influenza vaccine.

[b]Includes infants from Phase 2b weighing <5 kg and the full MELODY enrollment cohort.

Abbreviations: AE, adverse event; wGA, weeks gestational age.

**Table S4:** Treatment-Related AEs by System Organ Class and Preferred Term in Infants Entering Their First RSV Season Through 360 Days Post-Dose

| | Healthy Term and Preterm Infants Born ≥29 wGA[a] | | Infants Eligible for Palivizumab Entering Their First RSV Season | | | |
|---|---|---|---|---|---|---|
| | | | Preterm Infants Born ≤35 Weeks 0 days GA | | Infants With CHD/CLD | |
| **AEs, *n* (%)** | **Nirsevimab (*n* = 2570)** | **Placebo (*n* = 1284)** | **Nirsevimab (*n* = 406)** | **Palivizumab (*n* = 206)** | **Nirsevimab (*n* = 208)** | **Palivizumab (*n* = 98)** |
| Any treatment-related AE | 33 (1.3) | 18 (1.4) | 6 (1.5) | 4 (1.9) | 4 (1.9) | 2 (2.0) |
| Blood and lymphatic system disorders | 0 | 2 (0.2) | 0 | 0 | 0 | 0 |
| Anemia | 0 | 1 (<0.1) | 0 | 0 | 0 | 0 |
| Neutropenia | 0 | 1 (<0.1) | 0 | 0 | 0 | 0 |
| Gastrointestinal disorders | 2 (<0.1) | 2 (0.2) | 0 | 1 (0.5) | 0 | 0 |
| Diarrhea | 2 (<0.1) | 0 | 0 | 1 (0.5) | 0 | 0 |
| Constipation | 0 | 1 (<0.1) | 0 | 0 | 0 | 0 |
| Vomiting | 0 | 1 (<0.1) | 0 | 1 (0.5) | 0 | 0 |
| General disorders and administration site conditions | 7 (0.3) | 4 (0.3) | 0 | 2 (1.0) | 1 (0.5) | 1 (1.0) |
| Pyrexia | 3 (0.1) | 3 (0.2) | 0 | 2 (1.0) | 1 (0.5) | 0 |
| Injection site pain | 2 (<0.1) | 0 | 0 | 0 | 0 | 0 |
| Decreased activity | 1 (<0.1) | 0 | 0 | 0 | 0 | 0 |
| Injection site swelling | 1 (<0.1) | 0 | 0 | 0 | 0 | 0 |
| Fever neonatal | 0 | 1 (<0.1) | 0 | 0 | 0 | 0 |
| Injection site induration | 0 | 0 | 0 | 0 | 0 | 1 (1.0) |
| Infections and infestations | 1 (<0.1) | 2 (0.2) | 0 | 0 | 0 | 0 |
| Gastroenteritis | 1 (<0.1) | 0 | 0 | 0 | 0 | 0 |
| Pharyngitis | 0 | 1 (<0.1) | 0 | 0 | 0 | 0 |
| Upper respiratory tract infection | 0 | 1 (<0.1) | 0 | 0 | 0 | 0 |
| Injury, poisoning and procedural complications | 0 | 1 (<0.1) | 0 | 0 | 0 | 0 |
| Vaccine complication | 0 | 1 (<0.1) | 0 | 0 | 0 | 0 |
| Investigations | 1 (<0.1) | 2 (0.2) | 2 (0.5) | 0 | 0 | 0 |
| Body temperature increased | 0 | 0 | 2 (0.5) | 0 | 0 | 0 |
| Transaminases increased | 1[b] (<0.1) | 0 | 0 | 0 | 0 | 0 |
| Neutrophil count decreased | 0 | 2 (0.2) | 0 | 0 | 0 | 0 |
| Metabolism and nutrition disorders | 1 (<0.1) | 0 | 0 | 0 | 0 | 0 |
| Decreased appetite | 1 (<0.1) | 0 | 0 | 0 | 0 | 0 |
| Musculoskeletal and connective tissue disorders | 0 | 0 | 1 (0.2) | 0 | 0 | 0 |
| Myosclerosis[c] | 0 | 0 | 1[b] (0.2) | 0 | 0 | 0 |

| | | | | | | |
|---|---|---|---|---|---|---|
| Nervous system disorders | 2 (<0.1) | 1 (<0.1) | 0 | 0 | 0 | 0 |
| Hypersomnia | 1 (<0.1) | 0 | 0 | 0 | 0 | 0 |
| Somnolence | 1 (<0.1) | 1 (<0.1) | 0 | 0 | 0 | 0 |
| Psychiatric disorders | 4 (0.2) | 3 (0.2) | 2 (0.5) | 0 | 2 (1.0) | 1 (1.0) |
| Agitation | 0 | 0 | 1 (0.2) | 0 | 2 (1.0) | 0 |
| Irritability | 4 (0.2) | 3 (0.2) | 1 (0.2) | 0 | 0 | 1 (1.0) |
| Respiratory, thoracic and mediastinal disorders | 1 (<0.1) | 1 (<0.1) | 0 | 0 | 0 | 0 |
| Nasal congestion | 1 (<0.1) | 1 (<0.1) | 0 | 0 | 0 | 0 |
| Skin and subcutaneous tissue disorders | 15 (0.6) | 4 (0.3) | 1 (0.2) | 1 (0.5) | 1 (0.5) | 0 |
| Rash maculopapular | 7 (0.3) | 0 | 1 (0.2) | 0 | 0 | 0 |
| Rash | 3 (0.1) | 0 | 0 | 0 | 1 (0.5) | 0 |
| Dermatitis | 1 (<0.1) | 0 | 0 | 0 | 0 | 0 |
| Drug eruption | 1 (<0.1) | 0 | 0 | 0 | 0 | 0 |
| Petechiae | 1 (<0.1) | 0 | 0 | 0 | 0 | 0 |
| Rash papular | 1 (<0.1) | 1 (<0.1) | 0 | 0 | 0 | 0 |
| Skin hypopigmentation | 1 (<0.1) | 0 | 0 | 0 | 0 | 0 |
| Eczema | 0 | 1 (<0.1) | 0 | 0 | 0 | 0 |
| Rash macular | 0 | 0 | 0 | 1 (0.5) | 0 | 0 |

Participants with multiple events in the same preferred term were counted once in each of those preferred terms. Participants with events in more than one preferred term were counted once in each of those preferred terms.

[a]Includes infants from Phase 2b weighing <5kg and the full MELODY enrollment cohort.

[b]Mild (Grade 1) severity elevation of ALT and AST with return to normal reference range on a subsequent laboratory evaluation during the study.

[c]Event was reported by the investigator as "induration of the right thigh (muscular)" at site of injection of treatment and was inadvertently coded to the incorrect MedDRA Preferred Term of myosclerosis rather than injection site induration.

Abbreviations: AE, adverse event; ALT, alanine aminotransferase; AST, aspartate aminotransferase; CHD, congenital heart disease; CLD, congenital lung disease; GA, gestational age; RSV, respiratory syncytial virus; wGA, weeks gestational age.

**Table S5**: Nirsevimab Recipients with Post-Baseline Antidrug Antibodies Through 360 Days Post-Dose

| | Healthy Term and Preterm Infants Born ≥29 wGA[a] | Infants Eligible for palivizumab[b] | Children with CHD/CLD Entering Their Second RSV Season[c] | |
|---|---|---|---|---|
| **ADA Result** | **Nirsevimab (*n* = 2570)** | **Nirsevimab (*n* = 614)** | **Nirsevimab/Nirsevimab (*n* = 180)** | **Palivizumab/Nirsevimab (*n* = 40)[f]** |
| Participants with any post-baseline ADA to nirsevimab, % (*n*/*N*) | 6.2% (155/2493) | 5.8% (34/587)[d] | 11.7% (21/180)[e] | 2.5% (1/40) |

[a]Includes infants from Phase 2b weighing <5kg and the full MELODY enrollment cohort.
[b]Includes infants with CHD/CLD and premature infants born ≤35 weeks 0 days GA without CHD/CLD.
[c]Before the second season, children with CHD/CLD randomized to nirsevimab in the first season received a single IM dose of 200 mg nirsevimab followed by 4x once-monthly IM doses of placebo (nirsevimab/nirsevimab) and those randomized to palivizumab in the first season were re-randomized 1:1 to either a single IM dose of 200 mg nirsevimab followed by 4x once-monthly IM doses of placebo (palivizumab/nirsevimab) or 5x once-monthly IM doses of palivizumab (15 mg/kg per dose; palivizumab/palivizumab).
[d]24 participants in the preterm cohort and 10 in the CHD/CLD cohort had any post-baseline ADA to nirsevimab.
[e]Reflects the number of participants who had any post-baseline ADA to nirsevimab in Season 1 or Season 2 (baseline defined as prior to Season 1 dose). Of the 180 participants with CHD/CLD who received nirsevimab in 2 consecutive seasons, ADA were detected in 8/180 (4.4%) participants through Day 361 of Season 1. Only one participant had detectable ADA in both Season 1 and Season 2, showing that the second nirsevimab dose (Season 2 Day 1) did not boost the immune response to nirsevimab. At Day 31 and Day 151 of Season 2, ADA were detected in 1/90 (1.1%) and 0/168 (0.0%) participants, respectively, showing that there was no immune priming in participants who received a prior nirsevimab dose.
[f]Participants in the palivizumab/nirsevimab group were evaluated for post-baseline ADA to nirsevimab in Season 2 with baseline defined as prior to the Season 2 nirsevimab dose (either Season 1 Day 361 or Season 2 Day 1).
Abbreviations: ADA, antidrug antibodies; CHD, congenital heart disease; CLD, chronic lung disease of prematurity; IM, intramuscular; RSV, respiratory syncytial virus; wGA, weeks gestational age.

**Table S6:** AEs by Post-Baseline Antidrug Antibody Status Through 360 Days Post-Dose

| ADA Status, *n* (%) | **Healthy Term and Preterm Infants Born ≥29 wGA[a]** | | **Infants Eligible for Palivizumab Entering Their First RSV Season[b]** | |
|---|---|---|---|---|
| | **Nirsevimab (*n* = 2570)** | **Placebo (*n* = 1284)** | **Nirsevimab (*n* = 614)** | **Palivizumab (*n* = 304)** |
| AEs by post-baseline ADA status | | | | |
| Post-baseline ADA positive with at least 1 AE | 143/155 (92.3%) | 21/24 (87.5%) | 27/34 (79.4%) | 15/20 (75.0%) |
| Post-baseline ADA negative with at least 1 AE | 2062/2415 (85.4%) | 1067/1260 (84.7%) | 417/580 (71.9%) | 200/284 (70.4%) |
| Serious AEs[c] by post-baseline ADA status | | | | |
| Post-baseline ADA positive with at least 1 serious AE | 20/155 (12.9%) | 2/24 (8.3%) | 8/34 (23.5%) | 1/20 (5.0%) |
| Post-baseline ADA negative with at least 1 serious AE | 199/2415 (8.2%) | 141/1260 (11.2%) | 72/580 (12.4%) | 37/284 (13.0%) |

[a]Includes infants from Phase 2b weighing <5 kg and the full MELODY enrollment cohort.
[b]Includes infants with CHD/CLD and premature infants born ≤35 wGA without CHD/CLD.
[c]Defined as death, life-threatening, requiring inpatient hospitalization, prolongation of existing hospitalization, persistent or significant disability/incapacity, important medical event, congenital anomaly/birth defect.
Abbreviations: ADA, antidrug antibody; AE, adverse event; AESI, adverse event of special interest; CHD, congenital heart disease; CLD, chronic lung disease of prematurity; RSV, respiratory syncytial virus; wGA, weeks gestational age.

**Table S7:** AEs by Post-Baseline Antidrug Antibody Status in Children with CHD/CLD Entering Their Second RSV Season Through 360 Days Post-Dose

| | **Children with CHD/CLD Entering Their Second RSV Season** | | |
|---|---|---|---|
| **ADA Status, *n* (%)** | **Nirsevimab/nirsevimab (*n* = 180)** | **Palivizumab/nirsevimab (*n* = 40)** | **Palivizumab/palivizumab (*n* = 42)** |
| AEs by post-baseline ADA status | | | |
| Post-baseline ADA positive with at least 1 AE | 15/21 (71.4) | 1/1 (100.0) | 5/6 (83.3) |
| Post-baseline ADA negative with at least 1 AE | 115/159 (72.3) | 30/39 (76.9) | 24/36 (66.7) |
| Serious AEs by post-baseline ADA status | | | |
| Post-baseline ADA positive with at least 1 serious AE | 1/21 (4.8) | 0/1 (0.0) | 0/6 (0.0) |
| Post-baseline ADA negative with at least 1 serious AE | 22/159 (13.8) | 4/39 (10.3) | 2/36 (5.6) |

[a]Includes infants from Phase 2b weighing <5 kg and the full MELODY enrollment cohort.
[b]Includes infants with CHD/CLD and premature infants born ≤35 wGA without CHD/CLD.
[c]Defined as death, life-threatening, requiring inpatient hospitalization, prolongation of existing hospitalization, persistent or significant disability/incapacity, important medical event, congenital anomaly/birth defect.
Abbreviations: ADA, antidrug antibody; AE, adverse event; AESI, adverse event of special interest; CHD, congenital heart disease; CLD, chronic lung disease of prematurity; RSV, respiratory syncytial virus; wGA, weeks gestational age.

**Table S8:** Selected AEs Typically Solicited in Vaccine Studies in Infants within 7 Days Post-Dose in Healthy Term and Preterm Infants Born ≥29 wGA[a]

| **AE, *n* (%)** | **Nirsevimab (*n* = 2570)** | **Placebo (*n* = 1284)** |
|---|---|---|
| Pyrexia | 12 (0.5) | 8 (0.6) |
| Injection site reactions[b] | 7 (0.3) | 0 |
| Vomiting | 6 (0.2) | 4 (0.3) |
| Irritability | 5 (0.2) | 5 (0.4) |
| Somnolence | 2 (<0.1) | 2 (0.2) |
| Body temperature increased | 1 (<0.1) | 0 |
| Decreased appetite | 1 (<0.1) | 0 |

Participants with multiple events in the same preferred term were counted once in each of those preferred terms. Participants with events in more than one preferred term were counted once in each of those preferred terms.

[a]Includes infants from Phase 2b weighing <5 kg and the full MELODY enrollment cohort.

[b]Grouped term including preferred terms of injection site pain, injection site induration, and injection site swelling.

Abbreviations: AE, adverse event; wGA, weeks gestational age.

**Table S9**: Serious AEs[a] by System Organ Class and Preferred Term in Children with CHD/CLD Entering Their Second RSV Season[b] Through 360 Days Post-Dose

| | **Children with CHD/CLD Entering Their Second RSV Season** | | |
|---|---|---|---|
| **Serious AEs, *n* (%)** | **Nirsevimab/nirsevimab *n* = 180** | **Palivizumab/nirsevimab *n* = 40** | **Palivizumab/palivizumab *n* = 42** |
| 1 or more events | 23 (12.8) | 4 (10.0) | 2 (4.8) |
| Cardiac disorders | 1 (0.6) | 0 | 0 |
| Arrhythmia | 1 (0.6) | 0 | 0 |
| Congenital, familial, and genetic disorders | 1 (0.6) | 0 | 0 |
| Fallots tetralogy | 1[c] (0.6) | 0 | 0 |
| Gastrointestinal disorders | 2 (1.1) | 0 | 0 |
| Duodenal ulcer | 1 (0.6) | 0 | 0 |
| Intestinal obstruction | 1 (0.6) | 0 | 0 |
| Infections and infestations | 16 (8.9) | 4 (10.0) | 2 (4.8) |
| Bronchitis viral | 3 (1.7) | 0 | 0 |
| Gastroenteritis | 3 (1.7) | 1 (2.5) | 1 (2.4) |
| COVID-19 | 2 (1.1) | 0 | 0 |
| LRTI | 2 (1.1) | 1 (2.5) | 0 |
| Pneumonia | 2 (1.1) | 2 (5.0) | 0 |
| Bronchiolitis | 1 (0.6) | 0 | 0 |
| Meningitis aseptic | 1 (0.6) | 0 | 0 |
| Pharyngitis | 1 (0.6) | 0 | 0 |
| Rotavirus infection | 1 (0.6) | 0 | 0 |
| Upper respiratory tract infection | 1 (0.6) | 0 | 0 |
| Viral upper respiratory tract infection | 1 (0.6) | 0 | 0 |
| Bone abscess | 0 | 1 (2.5) | 0 |
| Ear infection | 0 | 1 (2.5) | 0 |
| Gastrointestinal infection | 0 | 1 (2.5) | 0 |
| LRTI viral | 0 | 0 | 1 (2.4) |
| Mastoiditis | 0 | 1 (2.5) | 0 |
| Otitis media | 0 | 1 (2.5) | 0 |
| Otitis media acute | 0 | 1 (2.5) | 0 |
| Investigations | 1 (0.6) | 0 | 0 |
| Catheterization cardiac | 1 (0.6) | 0 | 0 |
| Metabolism and nutrition disorders | 1 (0.6) | 0 | 0 |
| Failure to thrive | 1 (0.6) | 0 | 0 |
| Nervous system disorders | 1 (0.6) | 1 (2.5) | 0 |
| Syncope | 1 (0.6) | 0 | 0 |

| | | | |
|---|---|---|---|
| Nystagmus | 0 | 1 (2.5) | 0 |
| Renal and urinary disorders | 1 (0.6) | 0 | 0 |
| Calculus urinary | 1 (0.6) | 0 | 0 |
| Respiratory, thoracic, and mediastinal disorders | 2 (1.1) | 0 | 0 |
| Pleural effusion | 2 (1.1) | 0 | 0 |
| Vascular disorders | 1 (0.6) | 0 | 0 |
| Cyanosis | 1 (0.6) | 0 | 0 |

Participants with multiple events in the same category were counted once in that category; participants with events in >1 category were counted once in each category.
[a]Defined as death, life-threatening, requiring inpatient hospitalization, prolongation of existing hospitalization, persistent or significant disability/incapacity, important medical event, congenital anomaly/birth defect.
[b]Before the second season, children with CHD/CLD randomized to nirsevimab in the first season received a single IM dose of 200 mg nirsevimab followed by 4x once-monthly IM doses of placebo (nirsevimab/nirsevimab) and those randomized to palivizumab in the first season were re-randomized 1:1 to either a single IM dose of 200 mg nirsevimab followed by 4x once-monthly IM doses of placebo (palivizumab/nirsevimab) or 5x once-monthly IM doses of palivizumab (15 mg/kg per dose; palivizumab/palivizumab).
[c]Cardiac surgery for Tetralogy of Fallot.
Abbreviations: AE, adverse event; CHD, congenital heart disease; CLD, chronic lung disease of prematurity; COVID-19, coronavirus disease 2019; IM, intramuscular; LRTI, lower respiratory tract infection; RSV, respiratory syncytial virus.

**Table S10**: Treatment-Emergent AEs of Grade 3 or Greater Severity[a] by System Organ Class and Preferred Term in Children with CHD/CLD Entering Their Second RSV Season Through 360 Days Post-Dose

| | Children with CHD/CLD Entering Their Second RSV Season[b] | | |
|---|---|---|---|
| **AE of Grade 3 or Greater Severity, *n* (%)** | **Nirsevimab/nirsevimab *n* = 180** | **Palivizumab/nirsevimab *n* = 40** | **Palivizumab/palivizumab *n* = 42** |
| 1 or more events | 19 (10.6) | 4 (10.0) | 2 (4.8) |
| Cardiac disorders | 2 (1.1) | 0 | 0 |
| Arrhythmia | 1 (0.6) | 0 | 0 |
| Atrioventricular block | 1 (0.6) | 0 | 0 |
| Cardiac failure | 1 (0.6) | 0 | 0 |
| Congenital, familial, and genetic disorders | 1 (0.6) | 0 | 0 |
| Fallots tetralogy | 1[c] (0.6) | 0 | 0 |
| Gastrointestinal disorders | 2 (1.1) | 0 | 0 |
| Duodenal ulcer | 1 (0.6) | 0 | 0 |
| Intestinal obstruction | 1 (0.6) | 0 | 0 |
| General disorders and administration site conditions | 1 (0.6) | | |
| Pyrexia | 1 (0.6) | | |
| Infections and infestations | 13 (7.2) | 4 (10.0) | 2 (4.8) |
| Viral upper respiratory tract infection | 3 (1.7) | 1 (2.5) | 0 |
| Pneumonia | 3 (1.7) | 0 | 0 |
| Bronchitis viral | 2 (1.1) | 0 | 0 |
| Gastroenteritis | 2 (1.1) | 0 | 1 (2.4) |
| COVID-19 | 2 (1.1) | 0 | 0 |
| LRTI | 2 (1.1) | 1 (2.5) | 0 |
| Meningitis aseptic | 1 (0.6) | 0 | 0 |
| Nasopharyngitis | 1 (0.6) | 0 | 0 |
| Pharyngitis | 1 (0.6) | 0 | 0 |
| Rotavirus infection | 1 (0.6) | 0 | 0 |
| Upper respiratory tract infection | 1 (0.6) | 0 | 0 |
| Human herpesvirus 6 infection | 1 (0.6) | 0 | 0 |
| Urinary tract infection | 1 (0.6) | 0 | 0 |
| Bone abscess | 0 | 1 (2.5) | 0 |
| Ear infection | 0 | 1 (2.5) | 0 |
| Gastrointestinal infection | 0 | 1 (2.5) | 0 |
| LRTI viral | 0 | 0 | 1 (2.4) |
| Mastoiditis | 0 | 1 (2.5) | 0 |

| | | | |
|---|---|---|---|
| Otitis media acute | 0 | 2 (5.0) | 0 |
| Investigations | 1 (0.6) | 0 | 0 |
| Catheterization cardiac | 1 (0.6) | 0 | 0 |
| Nervous system disorders | 2 (1.1) | 0 | 0 |
| Ataxia | 1 (0.6) | 0 | 0 |
| Syncope | 1 (0.6) | 0 | 0 |
| Respiratory, thoracic, and mediastinal disorders | 2 (1.1) | 0 | 0 |
| Pleural effusion | 2 (1.1) | 0 | 0 |
| Vascular disorders | 1 (0.6) | 0 | 0 |
| Cyanosis | 1 (0.6) | 0 | 0 |

Participants with multiple events in the same category were counted once in that category; participants with events in >1 category were counted once in each category.
[a]Grade 1: mild, Grade 2: moderate; Grade 3: severe; Grade 4: life-threatening; Grade 5: fatal.
[b]Before the second season, children with CHD/CLD randomized to nirsevimab in the first season received a single IM dose of 200 mg nirsevimab followed by 4x once-monthly IM doses of placebo (nirsevimab/nirsevimab) and those randomized to palivizumab in the first season were re-randomized 1:1 to either a single IM dose of 200 mg nirsevimab followed by 4x once-monthly IM doses of placebo (palivizumab/nirsevimab) or 5x once-monthly IM doses of palivizumab (15 mg/kg per dose; palivizumab/palivizumab).
[c]Cardiac surgery for Tetralogy of Fallot.
Abbreviations: AE, adverse event; CHD, congenital heart disease; CLD, chronic lung disease of prematurity; COVID-19, coronavirus disease 2019; IM, intramuscular; LRTI, lower respiratory tract infection; RSV, respiratory syncytial virus.

**Supplementary Table S11.** Fatal Events in Healthy Term and Preterm Infants ≥29 wGA[a] Through 360 Days Post-Dose

| Trial | Demographics (Age at Randomization; Sex; Race; wGA at Birth) | Country | Study Day (age) at Death | Underlying Comorbidity | Cause of Death[b] |
|---|---|---|---|---|---|
| **Placebo group 0.2% (3/1284)** | | | | | |
| Phase 2b | 3.5 months; male; Black; 32 wGA | South Africa | 343 (~15 months) | Moderately preterm | **Pericardial effusion;** brought to hospital after mother found him not breathing. Died in hospital |
| Phase 2b | 1.1 months; female; Black; 30 wGA | South Africa | 26 (~2 months) | Very preterm; respiratory distress syndrome; Klebsiella sepsis (resolved prior to enrollment) | **Nosocomial pneumonia;** with contributing *E coli* meningitis |
| Phase 2b | 0.7 months; male; Black; 33 wGA | South Africa | 109 (~4 months) | Moderately preterm | **Pneumonia;** complicated by left-sided empyema; treated with traditional herbal medicine at home and was deceased on arrival to hospital |
| **Nirsevimab group 0.2% (6/2570)** | | | | | |
| Phase 2b | 3.9 months; female; Black; 31 wGA | South Africa | 123 (~8 months) | Very preterm | **Unknown;** mother reported infant was well when put to bed, found deceased in the morning; twin (nirsevimab group) completed study |
| Phase 2b | 1.9 months; male; White; 32 wGA | Estonia | 97 (~5 months) | Moderately preterm; respiratory distress syndrome | **Pulmonary vein stenosis**; unknown at study entry |
| MELODY | 3.1 months; male; Black; 37 wGA | South Africa | 143 (~8 months) | No underlying medical history | **Gastroenteritis;** No healthcare provider visit for the illness. Pronounced dead on arrival to emergency services. History of two prior episodes of gastroenteritis |
| MELODY | 6.8 months; female; Black; 38 wGA | South Africa | 338 (~18 months) | No underlying medical history | **Acute gastroenteritis;** No healthcare provider visit for the illness. Found lifeless after 2-3 days history of vomiting and diarrhea. Pronounced dead on arrival to emergency services |

| | | | | | |
|---|---|---|---|---|---|
| MELODY | 0.03 months; male; White; 40 wGA | Israel | 140 (~5 months) | No underlying medical history | **Unknown;** suspected undiagnosed chronic illness due to failure to thrive, AEs of recurrent vomiting, hypoglycemia, and anemia requiring transfusion |
| MELODY | 5.03 months; female; mixed race; 38 wGA | Panama | 286 (~14.5 months) | No underlying medical history | **Skull base fracture;** from automobile accident |

[a]Includes infants from Phase 2b weighing <5 kg and the full MELODY enrollment cohort.
[b]MedDRA Preferred Terms in bold
Abbreviations: AE, adverse event; MedDRA, Medical Dictionary for Regulatory Activities; wGA, weeks gestational age.

**Supplementary Table S12.** Fatal Events in Infants At Higher Risk of Severe RSV Disease[a] Through 360 Days Post-Dose

| MEDLEY Season | Demographics (Age at Randomization; Sex; Race; wGA) | Country | Study Day (age) at Death | Underlying Comorbidity | Cause of Death[b] |
|---|---|---|---|---|---|
| **Infants born ≤35 weeks 0 days GA without CHD/CLD – Palivizumab group 0% (0/206)** | | | | | |
| **Infants born ≤35 weeks 0 days GA without CHD/CLD – Nirsevimab group 0.5% (2/406)** | | | | | |
| Season 1 | 7.5 months; male; White; 29 wGA | Ukraine | 162 (~13 months) | Very preterm; Neonatal encephalopathy | Severe **COVID-19** pneumonia**,** admitted to ICU and died in the hospital |
| Season 1 | 1.2 months; male; White; 32 wGA | Bulgaria | 52 (~3 months) | Moderately preterm; Congenital Cytomegalovirus infection | **Bronchiolitis**, leading to acute cardiovascular and respiratory failure; severe protein calorie malnutrition at presentation |
| **Infants with CLD/CHD – Palivizumab group 1.0% (1/98)** | | | | | |
| Season 1 | 0.8 months; female; White; 38 wGA | Lithuania | 155 (~6 months) | CHD (ASD,[c] PDA); agenesis of the corpus callosum; cytogenic abnormality – feminine chromosomal mutation 46XX[11;22][q13.2;11.2]) | **Respiratory insufficiency** due to bronchiolitis |
| **Infants with CLD/CHD – Nirsevimab group 1.4% (3/208)** | | | | | |
| Season 1 | 2.8 months; female; White; 39 wGA | Hungary | 19 (~3 months) | CHD (partially corrected VSD,[c] ASD, coarctation of the aorta, PDA); dysgenesis of the corpus callosum; congenital cystic kidney disease | Sudden death due to **bronchopneumonia** |
| Season 1 | 2.3 months; female; other; 38 wGA | Mexico | 66 (~4.5 months) | CHD (VSD,[c] ASD); Trisomy 21; Hypothyroidism | **Cardiogenic shock** |
| Season 1 | 6.5 months; female; White; 38 wGA | Russian Federation | 19 (~3 months) | CHD (congenital pulmonary valve atresia with VSD,[c] MAPCA with unifocalization and creation of systemic pulmonary anastomosis); cerebral ischemia | **Cardiac failure**, pulmonary atresia |

[a]Additonal details on fatal events in the MEDLEY trial have been previously published (Domachowske JB, et al. *N Engl J Med* 2022; 386:892–894).
[b]MedDRA Preferred Terms in bold.
[c]Primary cardiac lesion as reported by the investigator.
Abbreviations: ASD, atrial septal defect; CHD, congenital heart disease; CLD, chronic lung disease of prematurity; COVID-19, coronavirus disease 2019; GA, gestational

age; ICU, intensive care unit; MAPCA, major aortopulmonary collateral arteries; MedDRA, Medical Dictionary for Regulatory Activities; PDA, patent ductus arteriosus; VSD, ventricular septal defect; wGA, weeks gestational age.
